# Development and Evaluation of the Abdominal Pain Knowledge Questionnaire (A-PKQ) for Children and Their Parents

**DOI:** 10.3390/children11070846

**Published:** 2024-07-12

**Authors:** Verena Neß, Clarissa Humberg, Franka Lucius, Leandra Eidt, Thomas Berger, Martin Claßen, Nils Christian Syring, Jens Berrang, Christine Vietor, Stephan Buderus, Lisa-Marie Rau, Julia Wager

**Affiliations:** 1 German Paediatric Pain Centre, Children’s and Adolescents’ Hospital Datteln, 45711 Datteln, Germany; v.ness@deutsches-kinderschmerzzentrum.de (V.N.); c.humberg@deutsches-kinderschmerzzentrum.de (C.H.); franka.lucius@uni-wh.de (F.L.); l.eidt@deutsches-kinderschmerzzentrum.de (L.E.); t.berger@kinderklinik-datteln.de (T.B.); l.rau@deutsches-kinderschmerzzentrum.de (L.-M.R.); 2 Department of Children’s Pain Therapy and Paediatric Palliative Care, Faculty of Health, School of Medicine, Witten/Herdecke University, 58455 Witten, Germany; 3 Hospital Group Gesundheit Nord, Klinikum Bremen Mitte, Centre for Children and Parents—Prof. Hess Paediatric Clinic, 28205 Bremen, Germany; m.classen@kinderarzt-schacht.de (M.C.); nilschristian.syring@gesundheitnord.de (N.C.S.); 4 Hospital Dortmund, Faculty of Health, School of Medicine, Witten/Herdecke University, 44137 Dortmund, Germany; jens.berrang@klinikumdo.de; 5 Techniker Krankenkasse, 22305 Hamburg, Germany; christine.vietor@tk.de; 6 GFO Clinics Bonn, St. Marienhospital Bonn, 53115 Bonn, Germany; stephan.buderus@gfo-kliniken-bonn.de

**Keywords:** functional abdominal pain, irritable bowel syndrome, knowledge, pediatric, parent, rasch analysis, item fit, bio-psycho-social, questionnaire, validation

## Abstract

Background: Abdominal pain is a common and often debilitating issue for children and adolescents. In many cases, it is not caused by a specific somatic condition but rather emerges from a complex interplay of bio-psycho-social factors, leading to functional abdominal pain (FAP). Given the complex nature of FAP, understanding its origins and how to effectively manage this condition is crucial. Until now, however, no questionnaire exists that targets knowledge in this specific domain. To address this, the Abdominal Pain Knowledge Questionnaire (A-PKQ) was developed. Methods: Two versions were created (one for children and one for parents) and tested in four gastroenterology clinics and one specialized pain clinic in Germany between November 2021 and February 2024. Children between 8 and 17 years of age (*N* = 128) and their accompanying parents (*N* = 131) participated in the study. Rasch analysis was used to test the performance of both versions of the questionnaire. Results: The original questionnaires exhibited good model and item fit. Subsequently, both questionnaires were refined to improve usability, resulting in final versions containing 10 items each. These final versions also demonstrated good model and item fit, with items assessing a variety of relevant domains. Conclusion: The A-PKQ is an important contribution to improving assessment in clinical trials focused on pediatric functional abdominal pain.

## 1. Introduction

Abdominal pain is one of the most common health conditions that children and adolescents experience. Younger children often experience acute abdominal pain—abdominal pain during everyday situations such as hunger, gas, or the urge to use the bathroom [1].

In many cases, however, abdominal pain does not occur acutely. In Western countries, up to 26% of children and adolescents are afflicted by recurrent abdominal pain [2,3]. When abdominal pain arises, many children and their parents suspect organic causes. However, it often originates from dysfunctional communication between the brain and gut [4]. This disruption in the gut–brain axis characterizes functional abdominal pain (FAP) [5,6]. FAP arises from a complex interplay of biological, social, and psychological factors and often significantly impairs the perceived quality of life of affected children and their parents [7,8]. The ROME IV diagnostic criteria define four subtypes of FAP: functional dyspepsia, abdominal migraine, irritable bowel syndrome, and functional abdominal pain not otherwise specified [9]. Importantly, FAP is diagnosed through exclusion, meaning that it is identified when other somatic conditions have been ruled out by a practitioner [10,11].

Understanding the origin of abdominal pain can be challenging for affected families, as it is not purely somatic [12]. Rather, a complex interplay of bio-psycho-social factors often triggers, exacerbates, and perpetuates this condition [13,14]. Children, for example, face a higher risk of developing FAP following a bacterial infection in the gastrointestinal tract [15,16]. Moreover, anxious children and those with behavioral problems are more often affected by FAP, as are those with anxious parents [17,18,19]. Addressing anxiety and psychological distress is crucial in treating FAP [20]. Unlike organic abdominal pain conditions, where rest is often the best treatment, customized activity is the best way to manage FAP and alleviate its symptoms [21,22].

As with many medical conditions, it is important to understand both the origins and sustaining factors of one’s condition and how to manage it effectively. Research in other areas of pain management has shown the significance of pain education in improving self-efficacy and management outcomes [23,24]. Knowledge plays a pivotal role in effective healthcare and is fundamental to Cognitive Behavioral Therapy (CBT), the recommended treatment for FAP [25,26].

There are existing questionnaires that assess patients’ knowledge about pain, primarily focusing on musculoskeletal pain in adults [27,28,29]. These questionnaires provide valuable tools to assess knowledge in these specific domains and examine variations in patients’ understanding of their condition. Utilizing a knowledge questionnaire is economical and easily manageable for patients, practitioners, and researchers. Patients need to invest only a reasonable amount of time, typically around their doctor’s visit [30]. Practitioners gain insights into their patients’ pre-existing knowledge, enabling them to build on this foundation and address gaps. Despite the importance of assessing knowledge in clinical and educational interventions, questionnaires assessing knowledge about pain for children and adolescents are rare. To our knowledge, no questionnaire exists that specifically tests the knowledge of children, adolescents, and their parents regarding abdominal pain, functional abdominal pain, and its proper management. 

In this study, we developed the Abdominal Pain Knowledge Questionnaire (A-PKQ) to assess the knowledge of patients and their parents about (functional) abdominal pain ((F)AP). We aimed to test the performance of newly developed items and determine whether they were model-compliant or needed to be excluded before finalizing the questionnaire. Items were also examined for redundancy and shortened if necessary. The questions were designed with varying levels of difficulty to ensure an even distribution of performance among patients and parents with different levels of knowledge.

## 2. Materials and Methods

### 2.1. Study Design 

The Abdominal Pain Knowledge Questionnaire (A-PKQ) was created as part of the project “Knowledge empowers! Empowerment of parent and child with functional abdominal pain”. A cornerstone of this project was the development of an educational website. The site comprehensively covers (1) general knowledge about the gastrointestinal tract, (2) abdominal pain, (3) functional abdominal pain, and (4) effective management of (F)AP, tailoring information to children, adolescents, and their parents. These areas were carefully chosen and refined based on recommendations from an expert committee. This committee included four pediatric gastroenterologists (T.B., J.B., M.C., and S.B.), healthcare professionals from the German Paediatric Pain Centre, an employee of a health insurance company (C.V.), and most importantly, children affected by FAP (*N* = 14) and their parents (*N* = 12). Two versions of the A-PKQ reflecting these domains were created to assess the knowledge of children and their parents regarding (F)AP and its proper management. Data for questionnaire validation was collected at a single measurement point.

### 2.2. Sample 

In total, *N* = 128 children and adolescents (60.2% female; *M*_age_ = 12.8, *SD* = 2.50) participated in our study. They answered an average of 18.9 (*SD* = 3.93) items out of the 20 in the questionnaire. The final analysis sample included *N* = 125 children and adolescents (61.6% female; *M*_age_ = 12.9, *SD* = 2.52) who answered at least two items of the A-PKQ, which was crucial for our analysis. Among the corresponding parents, *N* = 131 (80.5% female; *M*_age_ = 43.8, *SD* = 6.53) participated, answering an average of 17.0 (*SD* = 3.86) out of 18 items. A total of *N* = 128 parents (80.5% female; *M*_age_ = 43.8, *SD* = 6.53) were included in the analysis.

### 2.3. Development of the A-PKQ

Two versions of the A-PKQ were developed based on the content of our educational website, which covered (1) general knowledge about the gastrointestinal tract, (2) abdominal pain, (3) functional abdominal pain, and (4) effective management of (F)AP. For a brief overview, see Table 1 and Table 2; for the complete parent and child versions, see Supplementary Materials in Tables S1–S4. Questions regarding the gastrointestinal tract were included only in the child version, based on the assumption that parents already have basic knowledge of digestion and defecation and should not be made to feel that they are being tested on common knowledge. For children and adolescents, five questions were developed for each of the four website domains. Thus, the child version comprised twenty knowledge questions. For the parent version, six knowledge questions were developed for each of the three domains (abdominal pain, functional abdominal pain, and handling of (F)AP), resulting in 18 questions in total. The A-PKQ is designed to be engaging for both children and parents to prevent participants from losing interest in completing it or becoming distracted [31]. Questions in the child version were designed to be easily understood by younger children and were, therefore, also expected to be easily comprehensible for older patients as well. Instructions specified that if younger patients had difficulties with reading, parents were allowed to help with reading but were advised to refrain from giving hints about the correct answers.

### 2.4. Study Procedure

Patients and their parents were recruited as convenience samples at four pediatric gastroenterology clinics (Vestische Kinder- und Jugendklinik, 45711 Datteln, Germany; Klinikum Bremen Mitte, Eltern-Kind-Zentrum—Prof. Hess Kinderklinik, 28205 Bremen, Germany; Klinikum Dortmund, 44137 Dortmund, Germany; St. Marienhospital Bonn, 53115 Bonn, Germany) and one specialized pediatric pain clinic (German Paediatric Pain Centre, 45711 Datteln, Germany) in Germany. Recruitment at the four gastroenterology clinics took place between November 2021 and April 2022 (children: *N* = 42, parents: *N* = 48), and at the pain clinic between July 2022 and February 2024 (children: *N* = 86, parents: *N* = 83). All patients between 8 and 17 years of age presenting with abdominal pain and their parents were eligible. If one of these parameters was not fulfilled, patients could not be included in the study. Before appointments, clinic employees scanned the scheduled participants for eligible patients presenting with abdominal pain. Upon arrival at the clinic reception desk, eligible participants and their parents were informed about the questionnaire and asked individually for their interest in participating. Inclusion required that patients and parents provide assent and informed consent, respectively. Data collection took place immediately before their appointment. No incentives were given for participation. Refusals of study participation were mostly caused by time issues; not all patients arrived on time for their appointments, resulting in insufficient time for data collection. To avoid disrupting the clinic’s schedule, these families were not recruited. Participation in the study was only occasionally refused without a clear reason. Refusal reasons and incidence are not assessed systematically in this manuscript. Questionnaires were provided digitally on tablets. Besides the A-PKQ, the survey included demographics and questions about parental pain (“Do you have chronic pain, i.e., pain that has been recurring or persistent for at least 3 months?”; 0 = no, 1 = yes), as it was hypothesized that personal pain experiences might impact specific knowledge about abdominal pain. The knowledge questions were single-choice with four response options. The correct answers were not disclosed to the participants.

Ethics approval for the project was granted by the committee of Witten/Herdecke University (reference number 185/2020). Additional endorsements were obtained from the medical chamber of Bremen (application number 743), the Ethics Committee of the Medical Association of Westphalia-Lippe and Westfälische Wilhelms University of Münster (file number 2020-852-b-S), and the Medical Association of North Rhine (serial number: 2021140).

### 2.5. Statistical Analysis

Questionnaires were evaluated separately for patients and their parents. For the analysis, responses were coded binarily (1 = correct answer, 0 = wrong answer). In the present study, multiple-choice items were used, for which a three-parameter logistic (3PL) model is recommended in item response theory (IRT) [32]. However, model comparisons revealed no significant differences favoring a 2PL or 3PL model over a 1PL (i.e., Rasch) model. Therefore, Rasch modeling was used to evaluate the questions. In contrast to Classical Test Theory (CTT), which uses an underlying scaling model and relies on internal consistency checks, the probabilistic Rasch approach arranges items hierarchically (vertical scaling), calculating the probability of a correct response based on participant ability (estimated latent trait variable) and item difficulty [33]. Importantly, in IRT, item parameters remain constant when estimated in different samples (item invariance). Conditional maximum likelihood (CML) estimation was used to fit the Rasch model to the data. This approach tests the observed data against the dichotomous Rasch model to determine if the data conform to the model. Items fitting the Rasch model are expected to be consistent across different study populations. Misfit indices (infit and outfit) identify unexpected item residuals across participant responses. The chi-square statistic, normalized by degrees of freedom and reported as the mean-square statistic (MSQ), is used for evaluating item and person fit. An MSQ value of 1 indicates an ideal fit, while values between 0.5 and 1.5 are deemed acceptable for our sample size [34]. Values greater than 1 indicate items are less predictable by the model (i.e., underfitting), while values less than 1 indicate items are more predictable (i.e., overfitting). Infit and outfit statistics measure the discrepancies between expected and observed performance. Infit statistics are more sensitive to unexpected responses specific to the person (e.g., responses from idiosyncratic groups), whereas outfit statistics are more sensitive to outliers—significant discrepancies between item difficulty and a person’s ability (e.g., correct answers hit by chance). Moreover, person fit was assessed to determine if individual response patterns conform to the model, using standardized *t*-statistics ranging between 1.9 and −1.9 for reasonable predictability [35]. Andersen’s Likelihood Ratio Test (LRT) is utilized to confirm model fit with the data [36]. For this, data are split into two groups and tested for equality of item parameters. For the child version, data were split by gender; parent data was split by age using a median split at 44 years to accommodate its unbalanced gender distribution [37]. Parent data were also divided by chronic pain status to evaluate item parameters across groups (no chronic pain = 0, chronic pain = 1). Given the wide age range of child participants (8–17 years), acquired knowledge could vary significantly, especially since younger participants might have had challenges reading or understanding the questions. Thus, performance in older (≥13) and younger (<13) subsamples was investigated more closely. Moreover, the performance of each item was tested and evaluated using the Wald test to detect potential differential item functioning (DIF)—different item response patterns within subgroups despite them having the same ability level, like in the LRT [38]. Considering all the collected data, the A-PKQ was refined to retain the best-fitting items and remove those that did not fit the Rasch model or were redundant.

In addition to the formal evaluation of the questionnaire, feedback from patients and parents was collected informally through close communication with the staff conducting the study. Given that the questionnaire was newly designed, these verbal impressions provided valuable supplementary insights. However, due to the informal nature of this feedback, it is not presented as part of the formal results of this work. Nonetheless, the influence of this feedback on adjustments to the questionnaires is clearly stated in the results.

Descriptive statistics and the Rasch Model analyses were conducted using R and RStudio (Version 4.1.1; [39,40]). For the Rasch analyses, the eRm (version 1.0.2) package was used [41,42].

## 3. Results

### 3.1. Demographics and Descriptives

In total, *N* = 125 children and adolescents were included in the analysis, all of whom were born in Germany. All age-relevant German school types were represented (primary school: Grundschule = 5.5%; secondary school: Gymnasium = 31.2%; Gesamtschule = 16.4%; Realschule = 10.9%; Hauptschule = 10.2%; Förderschule = 9.4%; other = 16.4%). Among the parents that were included in the analysis (*N* = 128), 84.1% were born in Germany. Most parents were married to the birth parent of the child (69.2%), 10.5% were married to a new life partner, 10.5% were single, and 9.8% were in other types of relationships. Chronic pain was present in 38.3% of parents. Of all parents, 16.5% reported chronic back pain, 15.0% chronic headaches, 9.0% chronic leg pain, 6.8% chronic abdominal pain, 5.3% chronic arm pain, and 4.5% other types of chronic pain. 

### 3.2. Rasch Analysis Child Version

#### 3.2.1. Model Fit A-PKQ Child Version

The assumptions of unidimensionality and overall monotonicity were met. Mean-square statistics verified the compatibility of the items with the underlying Rasch model. Working with the cut-off values between 0.5 and 1.5 recommended by Wright and Linacre [34], only the item ‘warmth’ was not productive for measurement (Table 3). Testing for person-fit revealed five participants with unpredictable data [35]. Due to this small number representing < 5% of the sample, no adaptations were required by removing these participants.

**Figure 1:**
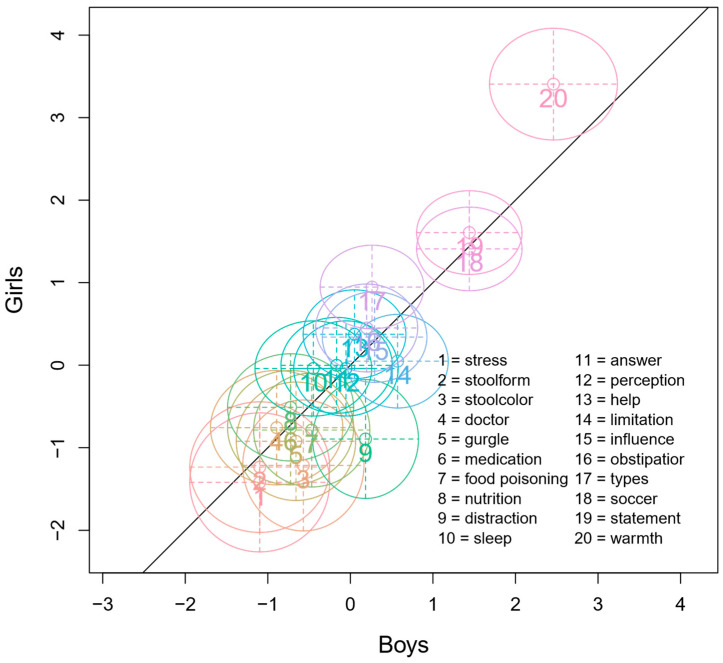
Visualization of Andersen’s Likelihood-Ratio Test (LRT) with a sample split by gender. On the *x*-axis, item parameters for boys are displayed, while the *y*-axis presents item parameters for girls. Zero indicates average difficulty, with items below zero being easier and those above being more difficult. The diagonal indicates parameter equality for girls and boys. Circles around items show confidence intervals (CI). CI’s overlapping with the diagonal indicate well-fitting items with high probabilities of parameter equality across groups. Notably, item 9 (‘distraction’) is significantly easier for girls than for boys.

The overall fit of the data to the Rasch model was investigated using Andersen’s LRT. For this, the data were split by gender to test the assumption that item parameters for each group are equivalent. Andersen’s LRT result was non-significant, meaning that item difficulties were similar across the two groups and fit the model well (χ^2^ (19) = 16.03, *p* = 0.656). A supporting graphical model is depicted in Figure 1, showing the model fit of item difficulties for girls and boys. Upon closer visual inspection of the graph, it appears that item 9 (‘distraction’) was more challenging for boys than for girls, as indicated by the confidence interval circle not intersecting the line denoting good fit. 

The Wald test was applied to test the fit of individual items to the model. Like the LRT test, this involved splitting the sample by gender. The results showed that samples differed significantly in terms of ‘distraction’ item difficulty (*z* = −2.19, *p* = 0.028), which was answered correctly more often by girls (see Table 4). This finding indicates the presence of DIF for the item ‘distraction’, meaning that girls and boys of similar ability responded differently to this particular item.

The examination of model fit was extended to compare overall model performance across two age groups (findings visualized in Figure S1). Andersen’s LRT was significant (χ^2^ (19) = 34.67, *p* = 0.015), indicating that item difficulties significantly differed between younger and older patients. The Wald test revealed DIF for the items ‘help’ (*z* = 2.18, *p* = 0.030), ‘gurgle’ (*z* = 2.58, *p* = 0.010), ‘food poisoning’ (*z* = −2.16, *p* = 0.031), and ‘soccer’ (*z* = 2.24, *p* = 0.025). Results indicated that younger patients generally answered these items correctly more often than older patients, except for the item ‘food poisoning’.

#### 3.2.2. Item Difficulty Analysis Child Version

**Figure 2:**
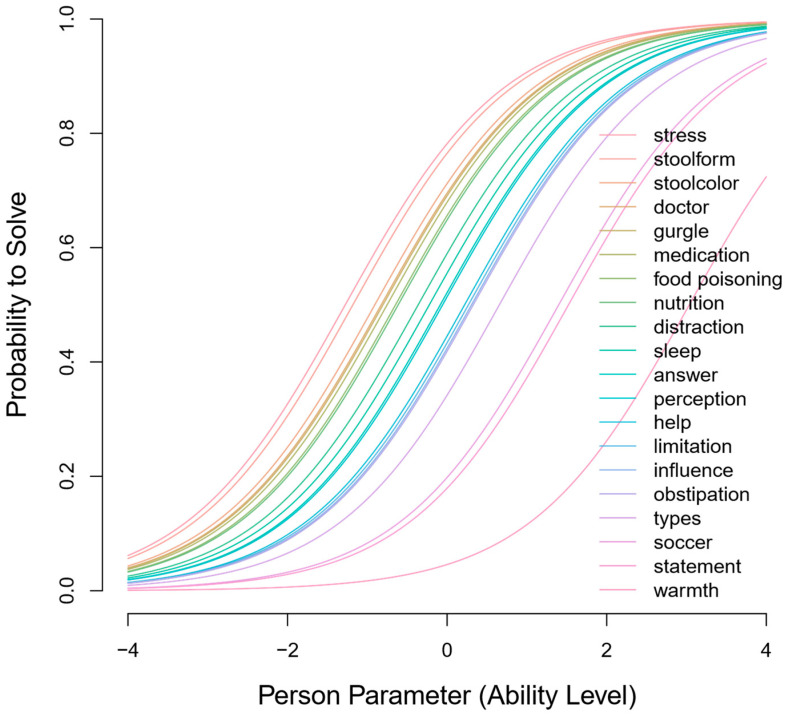
Item characteristic curve for the A-PKQ child version. The ICCs demonstrate the probability of answering an item correctly (*y*-axis) based on the person’s ability (*x*-axis). Negative values on the *x*-axis indicate lower ability, while positive values indicate higher ability. The difficulty of each item in the child A-PKQ is depicted in an individual ICC. Item names and colors are listed in the legend.

Item characteristic curves (ICC) were calculated for each item of the version for children and adolescents (Figure 2). These represent the relationship between the probability of answering an item correctly and the ability of the patient. For example, a participant with an ability parameter of 2 (*x*-axis) has approximately a 100% probability (*y*-axis) of solving the easiest items (e.g., ‘stool form’, ‘stress’) and a probability of around 25% of correctly answering the most difficult item, ‘warmth’. Visual inspection of the ICCs reveals that the items tend to be relatively easier than difficult, as indicated by the inflection points falling below zero on the *x*-axis.

Moreover, person-item maps were generated to illustrate the distribution of item difficulties relative to the abilities of all participants (Figure S2). Ideally, items should be distributed across the entire scale so that the range of participants’ abilities is matched to all item difficulty levels. The person-item map displays that several items are visually close to each other, indicating similar difficulty (Figure S2). Such clustering suggests that keeping all of these similar items in the questionnaire could lead to redundancy. 

#### 3.2.3. Modifying the A-PKQ Child Version

The Wald test revealed DIF for several items: ‘distraction’ showed differing levels of difficulty between girls and boys, while ‘gurgle’, ‘food poisoning’, ‘help’, and ‘soccer’ varied between younger and older patients. Consequently, these items were removed from the questionnaire. Additionally, the item ‘warmth’ was excluded due to its mean-squared fit statistics not meeting the predefined cut-off. Further analysis showed that several items had similar difficulty levels. As the length of the questionnaire was burdensome for many participants—especially younger patients, as reported by parents and study staff—the number of items was reduced while maintaining a variety of item difficulties. To minimize redundancy, ‘perception’ was removed from the questionnaire in favor of ‘answer’; ‘influence’ and ‘obstipation’ were removed in favor of ‘limitation’; and ‘stress’ was removed in favor of ‘stool form’.

**Figure 3:**
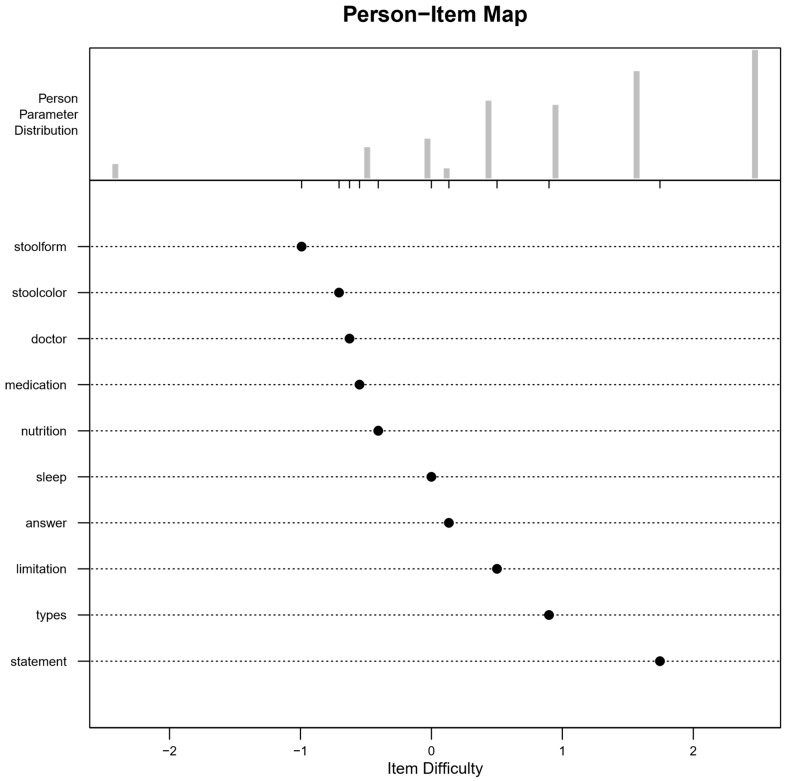
Person-item map of the final version of the child A-PKQ items. The *x*-axis denotes item difficulty and is adjusted to the range of individual abilities (−2 to 2). Five items are positioned below zero on the *x*-axis, indicating that patients with lower abilities also have a higher probability of solving this item correctly compared to items above zero. Two items are located around zero and are therefore suitable to identify patients with average ability. Three items are located above zero and are more likely to be answered correctly by patients with higher ability compared to patients with lower ability. The person parameter distribution reveals that the sample had generally high patient ability.

The following analysis of the remaining 10 items (Figure 3) showed that overall, the questionnaire still fit the underlying Rasch model when contrasting girls and boys (*χ*^2^ (9) = 6.80, *p* = 0.658). 

Also, model performance in the two age groups did not differ significantly (*χ*^2^ (9) = 9.22, *p* = 0.417). The Wald test showed no significant differences in item difficulties when comparing gender or age groups, confirming that no items exhibited DIF (Table S5). Infit and outfit statistics for all items were within the acceptable range of 0.5 and 1.5 (Table S6), and only three participants had misfitting responses. The final version of the child questionnaire covers a broad range of personal characteristics while minimizing the number of items. The person parameter distribution indicates that the sample had generally high ability (see Figure 3). The refined questionnaire consisted of ten items, five of which are easier (more likely to be answered correctly by patients with lower abilities, compared to items above zero), two are of moderate difficulty (suitable to identify patients with average abilities), and three are more difficult (aimed at those with higher abilities). The content includes three questions related to the ‘gastrointestinal tract’, two on ‘abdominal pain’, three on ‘functional abdominal pain’, and two focused on ‘abdominal pain management’.

### 3.3. Rasch Analysis Parent Version

#### 3.3.1. Model Fit A-PKQ Parent Version

The assumptions of unidimensionality and overall monotonicity were met. To test whether data conform to the underlying Rasch model, fit statistics were calculated using cut-off values for infit and outfit mean-squared statistics between 0.5 and 1.5, as recommended by Wright and Linacre [34]. The items ‘relaxation’ and ‘morbus colitis’ exceeded these defined cut-offs according to the outfit statistics (see Table 5). The examination of person-fit revealed that only four participants did not align with the model. Due to the small proportion of poorly fitting participants (<5%), it was not necessary to remove them from the analysis.

Andersen’s LRT was conducted to assess the overall fit of the data to the Rasch model. Given the differing proportions of mothers and fathers in the sample, it was not meaningful to test the equality of item parameters between these groups. Instead, parents were divided into two age groups using the median age of 44 years. Furthermore, due to the high number of parents with chronic pain, item parameter equality was also tested between parents experiencing chronic pain and those without. Andersen’s LRT indicated no significant differences in item difficulties between both age groups and chronic pain groups, suggesting a good fit with the model (age groups: *χ*^2^ (17) = 8.87, *p* = 0.944; pain groups: *χ*^2^ (17) = 14.56, *p* = 0.627). Graphical checks of model fit were also conducted using the distribution of item difficulties across age and pain groups (Supplementary Materials, Figures S3 and S4). All items were non-DIF, meaning that regardless of age group or pain group, participants of similar ability had equivalent probabilities of answering an item correctly. Item difficulties were further tested in the binary age and chronic pain groups using the Wald test. No statistically significant differences were found, and all items were non-DIF (Supplementary Materials, Table S7). 

#### 3.3.2. Item Difficulty Analysis Parent Version

**Figure 4:**
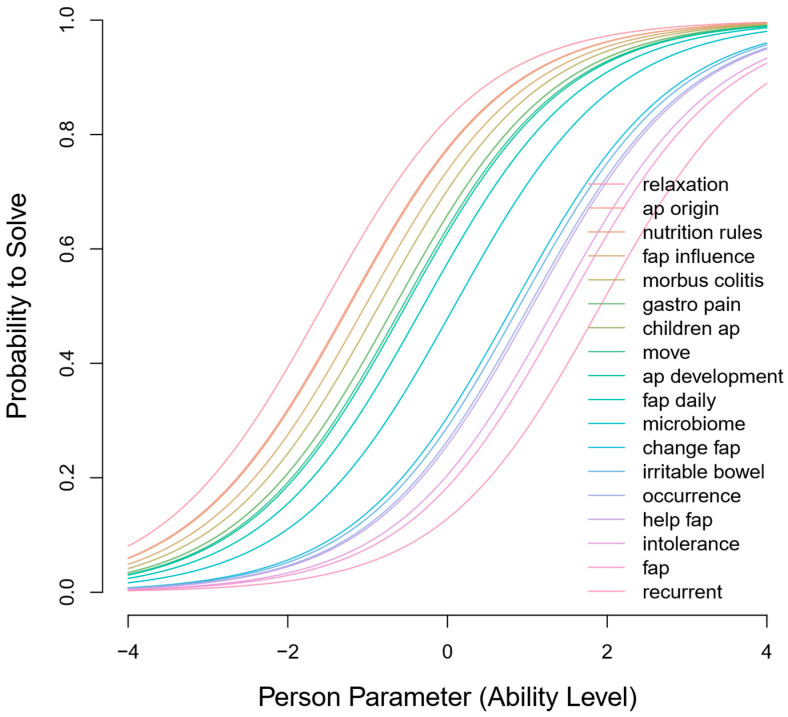
Item characteristic curve of A-PKQ Parent version. The ICCs demonstrate the probability of answering the item correctly (*y*-axis) given the person’s ability (*x*-axis). Negative values on the *x*-axis indicate lower ability, while positive values indicate higher ability. The difficulty of each item of the parent A-PKQ is represented by an individual ICC. The item names and colors are listed in the legend. For the overlapping items ‘children ap’ and ‘gastro pain’, item names are sequentially listed in the legend, with the color of ‘children ap’ prominently displayed.

For each item, an ICC assessed each item’s difficulty based on a person’s ability level (Figure 4).

Like the child version of the A-PKQ, most items in the parent version were easier to answer. The easiest item was ‘relaxation’, while ‘recurrent’ was the most difficult. Person-item maps displayed the full range of item difficulties across participants’ abilities (Figure S5). Again, several items were similarly difficult. Despite removing two items that did not contribute to the model, several other items produced redundancy. 

#### 3.3.3. Modifying the A-PKQ Parent Version

First, the two items, ‘relaxation’ and ‘morbus colitis’, were removed from the questionnaire because they did not fit the model well. Parents found the questionnaire too long, so it was shortened to ten items for practicability in clinical settings. The items ‘ap origin’, ‘fap influence’, ‘gastro pain’, ‘ap development’, and ‘irritable bowel’ were removed for redundancy, while ‘nutrition rules’ was removed for being too easy. We ensured the final questionnaire covered a broad range of items of varying difficulty for respondents of different abilities. 

**Figure 5:**
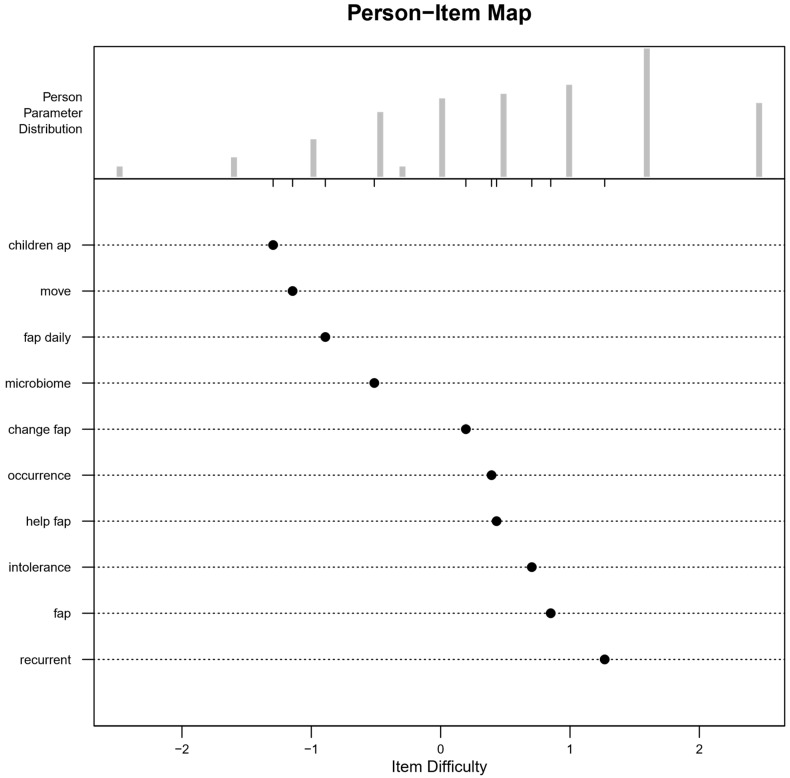
Person-item map of the parent version of the final 10 A-PKQ items. The final selection of items for the parent version of the A-PKQ shows six items positioned below zero on the *x*-axis and four items above zero. The person parameter distribution reveals a sample with generally higher abilities.

The final parent version of the A-PKQ fit the underlying model well (age groups: *χ*^2^ (9) = 3.86, *p* = 0.911; pain groups: *χ*^2^ (9) = 10.61, *p* = 0.303). All items were non-DIF, indicating uniform difficulty across the age and chronic pain subgroups, as confirmed by the Wald test (Supplementary Materials, Table S8). Infit and outfit statistics fell within the recommended cut-off values (Supplementary Materials, Table S9). Only one participant did not fit the model, but there was no need to remove him from the sample. Moreover, the questions were well-distributed across the person parameter distribution, with four questions likely to be answered correctly by parents with lower abilities compared to more difficult questions and six questions more likely to be answered correctly by parents with higher abilities compared to parents with lower abilities (Figure 5). The final version of the parent A-PKQ contained three questions on ‘abdominal pain’, three on ‘functional abdominal pain’, and four on ‘abdominal pain management’.

## 4. Discussion

The current study investigated the performance of the newly developed Abdominal Pain Knowledge Questionnaire (A-PKQ). While some multidimensional instruments for pain assessment in children and adolescents exist [43,44,45], knowledge is not typically included in these assessments. Two versions of the A-PKQ were created, one for children and adolescents (8–17 years of age) and another for parents. Both questionnaires were based on the domains of a new website about (functional) abdominal pain ((F)AP) (https://meine-bauchstelle.com/, accessed on 22 May 2024; English translation in progress). 

Statistical analyses guided modifications to the initial item pool, reducing the number of items from 20 in the child version and 18 in the parent version to 10 items each. These streamlined versions eliminate redundancy and accommodate differences in age, gender, and parental chronic pain status. Offering two 10-item questionnaires enables efficient data collection [46] and minimizes participant burden [46,47]. The final sets of items still cover all relevant domains presented online. 

The final versions of the knowledge questionnaires are suitable for use in secondary and tertiary care settings. In this study, we chose a diverse patient sample to ensure the questionnaires had broad applicability. It is expected, however, that patients visiting secondary care (e.g., gastroenterology clinics) may be less informed about their conditions compared to those in tertiary care (e.g., specialized pain clinics). This is because children and adolescents in tertiary care are likely to have had several appointments regarding their pain and received detailed information about their condition. Therefore, when employing the questionnaire in a more homogenous group, such as patients solely within secondary care, using less difficult items might improve application and the measurement of sensitivity to change. It is important to note that while the study’s questionnaire versions are designed for a broad patient sample, the analysis can also identify the most suitable items for a specific group. For example, if the questionnaire is applied to a population with generally lower personal ability, selecting easier items from our item pool is recommended. To facilitate this, our study data is available upon request to test the Rasch model with adjusted items.

Our study found that approximately 38% of participating parents suffered from chronic pain. Around 7% experienced chronic abdominal pain specifically. Previous works have highlighted that parental factors, including medical history, strongly influence a child’s development and experience of pain [48]. Other studies have revealed associations between parental chronic pain and chronic pain in their children [49,50]. While genetics may contribute to this association, environmental factors like parental behavior and pain catastrophizing have a noticeable adverse influence on the child’s pain outcomes [51,52]. At the time of data collection, the participants in our study were experiencing disabling abdominal pain and thus visited a secondary or tertiary care center. It is important to note that abdominal pain experienced in childhood often develops into recurrent abdominal pain and imposes more health restrictions during adulthood, which places a prospective burden on the healthcare sector and is thus crucial to address [17]. The high prevalence of chronic pain among these parents underscores the critical need for knowledge transfer about the condition and its proper management. However, our analysis revealed no difference in the accuracy of questionnaire responses between parents with and without chronic pain, suggesting that the need for information is similar across groups.

### 4.1. Strengths and Limitations

In our study, children as young as eight years old were included, as children at this age generally possess the appropriate cognitive development to complete a survey [53]. This assumption is supported by age group comparisons, which indicated that younger patients answered some items correctly more often than older patients. However, feedback from staff collecting the data revealed that younger children struggled with reading and understanding the questions, especially those that were text-heavy. Consequently, instructors or parents were encouraged to assist them with reading the questions if needed. It should be acknowledged, however, that having a parent read the questions to their child might bias the child’s answers [54]. The observed superiority of younger patients in answering some of the more text-heavy items might have resulted from parental assistance. Furthermore, since questionnaires were completed by parents and children simultaneously, and given that question categories overlap in both versions of the A-PKQ, parental assistance in reading the questions could have also impacted the parents’ answers on their own questionnaires. One potential solution for this could be to have study staff support younger participants instead of parents.

Another limitation of the study was the lack of systematic recording of the reasons and incidence of refusal to participate, which precluded further evaluation. Importantly, the final version of the child A-PKQ demonstrated a good fit to the Rasch model across a wide age range. Despite some parents reporting that the questionnaires were challenging, the overall low item difficulty contradicts this feedback. It is worth noting that a significant proportion of parents in this sample suffered from chronic pain, which may have influenced their perception of questionnaire difficulty due to their own high expectations of themselves or fear of failure—traits that are more prevalent among people with chronic pain [55]. Additionally, some parents may have doubted the accuracy of their answers, leading them to perceive the questions as more difficult. Their concerns regarding the difficulty of the child questionnaire might also reflect lower expectations of their child’s abilities, leading them to overestimate the questionnaire’s difficulty as a rationale for their child’s (falsely) anticipated poor performance [56]. Misjudgments by parents regarding their child’s condition have already been demonstrated in surveys, where parents underestimated their children’s symptoms of abdominal pain [57].

### 4.2. Practical Implications and Future Directions

The development of our knowledge questionnaire, linked to our educational website about (F)AP and its management, is a valuable tool for monitoring the course of abdominal pain treatment in young patients. The questionnaire is designed to be less time-consuming and impose a minimal burden on patients, making its application in clinical settings feasible with little effort required from medical staff. It offers practitioners quick and helpful insights into the patients’ and parents’ knowledge levels, which could contribute to more effective help during appointments. The questionnaires can be adapted to match the expected abilities of the respondents within a sample based on the data collected in this study. However, future studies should bear in mind the potential issues with parents reading study questions to their young children. Whenever possible, study staff should handle this task.

## 5. Conclusions

The present study demonstrates the successful development of the A-PKQ, a questionnaire designed to measure knowledge about (Functional) Abdominal Pain ((F)AP). The A-PKQ stands out as an important contribution to use in interventional studies aimed at pediatric (F)AP. Demonstrating robust psychometric properties, it serves as a useful tool to evaluate knowledge in research contexts, with the potential to also be used in clinical settings and pain education.

## Figures and Tables

**Table 1:** Short description of A-PKQ child version items.

	Short Item Description		Short Item Description
Gastrointestinal Tract		Functional Abdominal Pain	
gurgle	Origin of belly gurgling due to food processing	answer	Malfunctioning in the gut–brain signaling in functional abdominal pain (FAP)
stool color	Discoloration in the stool due to colored food	influence	Influencing factors for FAP
stool form	Normal variations in stool form and -consistency	limitation	FAP as an exclusion diagnosis
stress	Development of abdominal pain due to emotional stress and full schedule	soccer	Importance of distraction in FAP
statement	Alterations in the gastrointestinal tract due to emotions	medication	Lack of effectiveness of pain medication in FAP
Abdominal Pain		Handling FAP	
perception	Abdominal pain perception due to gut–brain interaction	help	Advantages of talking to parents about FAP
doctor	Importance of the doctor’s consultation to find the origin of abdominal pain	nutrition	Importance of a balanced diet in FAP
types	Acute and chronic pain types	warmth	Lack of lasting effectiveness of a hot water bottle in FAP
obstipation	Typical manifestation of obstipation	sleep	Positive mindset before sleep
food poisoning	Typical manifestation of food poisoning	distraction	Importance of distraction in FAP for the brain

**Table 2:** Short description of A-PKQ Parent Version items.

	Short Item Description		Short Item Description
Abdominal Pain		Functional Abdominal Pain Management	
ap development	Abdominal pain due to various structural and functional causes in the gastrointestinal tract	nutrition rules	Importance of a balanced diet in FAP
children ap	Abdominal pain causes in young children	occurrence	Checking potential causes of abdominal pain with a doctor
gastro pain	Handling of gastrointestinal infection	fap daily	Normal daily routine helps with FAP
recurrent	Development of recurrent abdominal pain	help fap	Importance of distraction in FAP
intolerance	Fructose- and Lactose intolerance	move	Physical activity improves FAP symptoms
morbus colitis	Chronic inflammatory bowel diseases	relaxation	Planned breaks are good but activity is just as important
Functional Abdominal Pain			
fap	Gut–brain communication due to FAP		
irritable bowel	Types of FAP like irritable bowel syndrome		
fap influence	Influencing factors for FAP		
microbiome	Role of the microbiome in FAP		
ap origin	Association between stress or negative emotions and FAP		
change fap	Drawing attention to the belly increases abdominal pain in FAP		

**Table 3:** Mean-square fit statistics for all items in the A-PKQ child version.

	Outfit (MSQ) ^1^	Infit (MSQ)		Outfit (MSQ)	Infit (MSQ)
Gastrointestinal tract			Functional Abdominal Pain		
gurgle	0.78	0.94	answer	0.76	0.95
stool color	0.58	0.88	influence	1.10	1.10
stool form	0.76	0.86	limitation	0.92	0.96
stress	0.94	0.96	soccer	0.98	0.97
statement	1.10	1.11	medication	0.92	0.95
Abdominal Pain			Handling FAP		
perception	0.93	1.00	help	1.01	1.01
doctor	1.26	1.07	nutrition	0.68	0.88
types	0.89	0.93	warmth	1.98 *	1.06
obstipation	1.19	1.05	sleep	0.73	0.91
food poisoning	0.83	0.94	distraction	0.83	0.94

* Items that exceeded the cut-off for item fit (0.5–1.5). ^1^ Mean-square fit statistics: measure the extent of distortion of the measuring system (1.0 is the expected value).

**Table 4:** Wald test results for item difficulty by gender and age.

	Gender		Age	
	*z*-Statistic	*p*-Value	*z*-Statistic	*p*-Value
Gastrointestinal tract				
gurgle	−0.51	0.613	2.58 *	0.010 *
stool color	−1.17	0.242	0.29	0.769
stool form	−0.23	0.817	−0.70	0.485
stress	−0.53	0.598	−1.10	0.273
statement	0.40	0.691	−0.91	0.366
Abdominal Pain				
perception	0.03	0.976	0.02	0.988
doctor	0.25	0.802	0.62	0.539
types	1.66	0.097	−1.54	0.124
obstipation	0.61	0.543	0.39	0.702
food poisoning	−0.63	0.527	−2.16 *	0.031 *
Functional Abdominal Pain				
answer	0.36	0.718	0.23	0.818
influence	0.12	0.914	−1.08	0.279
limitation	−1.23	0.219	1.22	0.224
soccer	−0.08	0.938	2.24 *	0.025 *
medication	−0.06	0.949	−0.76	0.449
Handling FAP				
help	0.77	0.440	2.18 *	0.030 *
nutrition	0.42	0.678	1.44	0.149
warmth	1.80	0.071	−1.40	0.161
sleep	0.88	0.381	0.78	0.434
distraction	−2.19 *	0.028 *	−0.56	0.576

Negative values indicate that the item is easier for girls compared to boys or for older compared to younger patients. * Statistically significant difference in item difficulty between the two groups, and thus a divergence from the model (*p* < 0.05).

**Table 5:** Mean-square fit statistics for all items in the A-PKQ parent version.

	Outfit (MSQ) ^1^	Infit (MSQ)		Outfit (MSQ)	Infit (MSQ)
Abdominal Pain			Functional Abdominal Pain Management		
ap development	1.06	0.91	nutrition rules	0.66	0.92
children ap	1.03	0.90	occurrence	1.29	1.24
gastro pain	1.06	0.93	fap daily	0.80	0.97
recurrent	1.31	1.20	help fap	0.82	0.86
intolerance	1.10	1.02	move	0.80	0.83
morbus colitis	0.49 *	0.84	relaxation	0.49 *	0.86
Functional Abdominal Pain					
fap	0.91	0.95			
irritable bowel	1.05	1.03			
fap influence	1.01	1.00			
microbiome	1.10	1.19			
ap origin	0.59	0.81			
change fap	0.79	0.84			

* Items that exceeded the cut-off for item fit (0.5–1.5). ^1^ Mean-square fit statistics: measure the extent of distortion of the measuring system (1.0 is the expected value).

## Data Availability

The data presented in this study are available on request from the corresponding author (J.W.). The data are not publicly available because, as described in the consent forms and thus, due to legal reasons, may only be analyzed with legitimate interest.

## Supplementary Materials

The following supporting information can be downloaded at: https://www.mdpi.com/article/10.3390/children11070846/s1, Table S1: Full A-PKQ child version—original German version; Table S2: Full A-PKQ child version—non-validated English translation; Table S3: Full A-PKQ parent version—original German version; Table S4: Full A-PKQ parent version—non-validated English translation; Table S5: Wald test results for the final item fit by comparing item difficulties by gender and age; Table S6: Mean-square fit statistics for the final ten items of the A-PKQ child version; Table S7: Wald test results for item fit by comparing parent item difficulties by age and chronic pain; Table S8: Wald test results for final item fit by comparing parent item difficulties by age and chronic pain; Table S9: Mean-square fit statistics for the final 10 items of the A-PKQ parent version; Figure S1: Visualization of Andersen’s Likelihood-Ratio Test (LRT) with the child sample split by age; Figure S2: Person-item map of the A-PKQ child version; Figure S3: Visualization of Andersen’s Likelihood-Ratio Test (LRT) with parent sample split by age group; Figure S4: Visualization of Andersen’s Likelihood-Ratio Test (LRT) with parent sample split by chronic pain group; Figure S5: Person-item map of the parent version of all A-PKQ items.

## References

[1] Hyman P. Bellyaches in Children. https://iffgd.org/wp-content/uploads/809-Bellyaches-in-Children-1.pdf.

[2] Chitkara D.K., Rawat D.J., Talley N.J. (2005). The epidemiology of childhood recurrent abdominal pain in Western countries: A systematic review. Am. J. Gastroenterol..

[3] Sjölund J., Uusijärvi A., Tornkvist N.T., Kull I., Bergström A., Alm J., Törnblom H., Olén O., Simrén M. (2021). Prevalence and Progression of Recurrent Abdominal Pain, From Early Childhood to Adolescence. Clin. Gastroenterol. Hepatol..

[4] Mayer E.A., Gupta A., Kilpatrick L.A., Hong J.-Y. (2015). Imaging brain mechanisms in chronic visceral pain. Pain.

[5] Di Lorenzo C., Colletti R.B., Lehmann H.P., Boyle J.T., Gerson W.T., Hyams J.S., Squires R.H., Walker L.S., Kanda P.T. (2005). Chronic Abdominal Pain In Children: A Technical Report of the American Academy of Pediatrics and the North American Society for Pediatric Gastroenterology, Hepatology and Nutrition. J. Pediatr. Gastroenterol. Nutr..

[6] Thornton G.C.D., Goldacre M.J., Goldacre R., Howarth L.J. (2016). Diagnostic outcomes following childhood non-specific abdominal pain: A record-linkage study. Arch. Dis. Child..

[7] Youssef N.N., Murphy T.G., Langseder A.L., Rosh J.R. (2006). Quality of life for children with functional abdominal pain: A comparison study of patients’ and parents’ perceptions. Pediatrics.

[8] Wager J., Zernikow B. (2014). Was ist Schmerz?. Monatsschr. Kinderheilkd..

[9] Hyams J.S., Di Lorenzo C., Saps M., Shulman R.J., Staiano A., van Tilburg M. (2016). Functional Disorders: Children and Adolescents. Gastroenterology.

[10] Brown L.K., Beattie R.M., Tighe M.P. (2016). Practical management of functional abdominal pain in children. Arch. Dis. Child..

[11] Korterink J., Devanarayana N.M., Rajindrajith S., Vlieger A., Benninga M.A. (2015). Childhood functional abdominal pain: Mechanisms and management. Nat. Rev. Gastroenterol. Hepatol..

[12] Brekke M., Brodwall A. (2020). Understanding parents’ experiences of disease course and influencing factors: A 3-year follow-up qualitative study among parents of children with functional abdominal pain. BMJ Open.

[13] Crushell E., Rowland M., Doherty M., Gormally S., Harty S., Bourke B., Drumm B. (2003). Importance of parental conceptual model of illness in severe recurrent abdominal pain. Pediatrics.

[14] Lindley K.J., Glaser D., Milla P.J. (2005). Consumerism in healthcare can be detrimental to child health: Lessons from children with functional abdominal pain. Arch. Dis. Child..

[15] Schwille-Kiuntke J., Unverdorben A., Weimer K., Schlarb A.A., Gulewitsch M.D., Ellert U., Enck P. (2015). Bacterial infections in childhood: A risk factor for gastrointestinal and other diseases?. United Eur. Gastroenterol. J..

[16] Thabane M., Simunovic M., Akhtar-Danesh N., Garg A.X., Clark W.F., Collins S.M., Salvadori M., Marshall J.K. (2010). An outbreak of acute bacterial gastroenteritis is associated with an increased incidence of irritable bowel syndrome in children. Am. J. Gastroenterol..

[17] Ayonrinde O.T., Ayonrinde O.A., Adams L.A., Sanfilippo F.M., O’ Sullivan T.A., Robinson M., Oddy W.H., Olynyk J.K. (2020). The relationship between abdominal pain and emotional wellbeing in children and adolescents in the Raine Study. Sci. Rep..

[18] Campo J.V., Bridge J., Ehmann M., Altman S., Lucas A., Birmaher B., Di Lorenzo C., Iyengar S., Brent D.A. (2004). Recurrent abdominal pain, anxiety, and depression in primary care. Pediatrics.

[19] Ramchandani P.G., Murray L., Romano G., Vlachos H., Stein A. (2011). An investigation of health anxiety in families where children have recurrent abdominal pain. J. Pediatr. Psychol..

[20] Iovino P., Tremolaterra F., Boccia G., Miele E., Ruju F.M., Staiano A. (2009). Irritable bowel syndrome in childhood: Visceral hypersensitivity and psychosocial aspects. Neurogastroenterol. Motil..

[21] Simons L.E., Claar R.L., Logan D.L. (2008). Chronic pain in adolescence: Parental responses, adolescent coping, and their impact on adolescent’s pain behaviors. J. Pediatr. Psychol..

[22] Puckett-Perez S., Gresl B. (2022). Psychological treatment for pediatric functional abdominal pain disorders. Curr. Opin. Pediatr..

[23] Ryan C.G., Gray H.G., Newton M., Granat M.H. (2010). Pain biology education and exercise classes compared to pain biology education alone for individuals with chronic low back pain: A pilot randomised controlled trial. Man. Ther..

[24] Ma X., Yu W., Lu Y., Yang H., Li X., Kang D. (2022). Pain knowledge of patients and family caregivers as predictors of pain management outcomes in cancer patients: A multicenter study in China. Support. Care Cancer.

[25] Layer P., Andresen V., Allescher H.-D., Bischoff S.C., Niesler B., Freitag M.H. (2021). Update S3-Leitlinie Reizdarmsyndrom: Definition, Pathophysiologie, Diagnostik und Therapie: Gemeinsame Leitlinie der Deutschen Gesellschaft für Gastroenterologie, Verdauungs- und Stoffwechselkrankheiten (DGVS) und der Deutschen Gesellschaft für Neurogastroenterologie und Motilität (DGNM)-Juni 2021-AWMF-Registriernummer: 021/016. Z. Gastroenterol..

[26] Löfgren E., Lindfors P., Nilsson K., Wannstedt J., Bonnert M., Uusijärvi A. (2024). Gastrointestinal Group Education for Children and Adolescents with Functional Abdominal Pain Disorders—A Feasibility Study of a Brief Intervention. Gastrointest. Disord..

[27] Maciel S.C., Jennings F., Jones A., Natour J. (2009). The development and validation of a Low Back Pain Knowledge Questionnaire-LKQ. Clinics.

[28] Hennell S.L., Brownsell C., Dawson J.K. (2004). Development, validation and use of a patient knowledge questionnaire (PKQ) for patients with early rheumatoid arthritis. Rheumatology.

[29] Hill J., Bird H. (2007). Patient knowledge and misconceptions of osteoarthritis assessed by a validated self-completed knowledge questionnaire (PKQ-OA). Rheumatology.

[30] Meadows K.A. (2003). So you want to do research? 5: Questionnaire design. Br. J. Community Nurs..

[31] Qiu S., Xia Y., Tian F., Yang Y., Song J., Chen L., Mei H., Jiang F., Bao N., Liu S. (2021). Using a cartoon questionnaire to improve consent process in children: A randomized controlled survey. Pediatr. Res..

[32] Nguyen T.H., Han H.-R., Kim M.T., Chan K.S. (2014). An introduction to item response theory for patient-reported outcome measurement. Patient.

[33] Rasch G. (1993). Probabilistic Models for Some Intelligence and Attainment Tests.

[34] Wright B.D., Linacre J.M. (1994). Reasonable mean-square fit values. Rasch Meas. Trans..

[35] Wright B.D., Linacre J.M. (2002). What do Infit and Outfit, Mean-square and Standardized mean?. Rasch Meas. Trans..

[36] Andersen E.B. (1973). A goodness of fit test for the rasch model. Psychometrika.

[37] Krammer G. (2018). The Andersen Likelihood Ratio Test with a Random Split Criterion Lacks Power. J. Mod. Appl. Stat. Methods.

[38] Wald A. (1943). Tests of statistical hypotheses concerning several parameters when the number of observations is large. Trans. Am. Math. Soc..

[39] R Core Team (2021). R: A Language and Environment for Statistical Computing.

[40] Posit Team (2023). RStudio: Integrated Development Environment for R.

[41] Mair P., Hatzinger R., Maier M.J., Rusch T., Debelak R. (2021). eRm: Extended Rasch Modeling. https://cran.r-project.org/web/packages/eRm/index.html.

[42] Mair P., Hatzinger R. (2007). Extended Rasch Modeling: The eRm Package for the Application of IRT Models in R. J. Stat. Soft..

[43] Schroeder S., Hechler T., Denecke H., Müller-Busch M., Martin A., Zernikow B. (2010). Deutscher Schmerzfragebogen für Kinder, Jugendliche und deren Eltern (DSF-KJ). Schmerz.

[44] Malaty H.M., Abudayyeh S., O’Malley K.J., Wilsey M.J., Fraley K., Gilger M.A., Hollier D., Graham D.Y., Rabeneck L. (2005). Development of a multidimensional measure for recurrent abdominal pain in children: Population-based studies in three settings. Pediatrics.

[45] Eccleston C., McCracken L.M., Jordan A., Sleed M. (2007). Development and preliminary psychometric evaluation of the parent report version of the Bath Adolescent Pain Questionnaire (BAPQ-P): A multidimensional parent report instrument to assess the impact of chronic pain on adolescents. Pain.

[46] Jeong D., Aggarwal S., Robinson J., Kumar N., Spearot A., Park D.S. (2023). Exhaustive or exhausting? Evidence on respondent fatigue in long surveys. J. Dev. Econ..

[47] Brown R.F., St John A., Hu Y., Sandhu G. (2024). Differential Electronic Survey Response: Does Survey Fatigue Affect Everyone Equally?. J. Surg. Res..

[48] Palermo T.M., Valrie C.R., Karlson C.W. (2014). Family and parent influences on pediatric chronic pain: A developmental perspective. Am. Psychol..

[49] Hoftun G.B., Romundstad P.R., Rygg M. (2013). Association of parental chronic pain with chronic pain in the adolescent and young adult: Family linkage data from the HUNT Study. JAMA Pediatr..

[50] Higgins K.S., Birnie K.A., Chambers C.T., Wilson A.C., Caes L., Clark A.J., Lynch M., Stinson J., Campbell-Yeo M. (2015). Offspring of parents with chronic pain: A systematic review and meta-analysis of pain, health, psychological, and family outcomes. Pain.

[51] Goodman J.E., McGrath P.J. (2003). Mothers’ modeling influences children’s pain during a cold pressor task. Pain.

[52] Wilson A.C., Moss A., Palermo T.M., Fales J.L. (2013). Parent Pain and Catastrophizing Are Associated with Pain, Somatic Symptoms, and Pain-Related Disability among Early Adolescents. J. Pediatr. Psychol..

[53] Borgers N., de Leeuw E., Hox J. (2000). Children as Respondents in Survey Research: Cognitive Development and Response Quality 1. Bull. Sociol. Methodol..

[54] Choi B.C., Pak A.W. (2005). A Catalog of Biases in Questionnaires. Prev. Chronic Dis..

[55] Molenaar B., Willems C., Verbunt J., Goossens M. (2021). Achievement Goals, Fear of Failure and Self-Handicapping in Young Elite Athletes with and without Chronic Pain. Children.

[56] Jaaniste T., Jia N., Lang T., Goodison-Farnsworth E.M., McCormick M., Anderson D. (2016). The relationship between parental attitudes and behaviours in the context of paediatric chronic pain. Child Care Health Dev..

[57] Saps M., Adams P., Bonilla S., Chogle A., Nichols-Vinueza D. (2012). Parental report of abdominal pain and abdominal pain-related functional gastrointestinal disorders from a community survey. J. Pediatr. Gastroenterol. Nutr..

