# Supplementary material for: Development and Evaluation of the Abdominal Pain Knowledge Questionnaire (A-PKQ) for Children and Their Parents
Source: Children (Basel). 2024 Jul 12;11(7):846. doi: 10.3390/children11070846 (PMC11276134; doi:10.3390/children11070846)
Supplement: Supplementary file 1 [file children-11-00846-s001.zip › children-3047342-supplementary.pdf]

Supplementary Material

Table of Contents

Table S1 2

Table S2 7

Table S3 11

Table S4 15

Table S5 19

Table S6 19

Table S7 20

Table S8 21

Table S9 22

Figure S1 23

Figure S2 24

Figure S3 26

Figure S4 27

Table S1

Table S1. Full A-PKQ Child Version – Original German version.

	Questions and response options[1]
Abbreviated Items[2]	
**stoolform**	**Welche Aussage zu Stuhlform und -konsistenz stimmt?**<br>**A:** Die Stuhlform ist immer mal wieder unterschiedlich. Am häufigsten kommt die Würstchenform vor. (2)[3]<br>**B:** Wenn der Stuhl sehr hart ist, soll man sich möglichst wenig bewegen. Dann wird er weicher und kommt leichter heraus. (3)<br>**C:** Sobald der Stuhlgang mal weicher oder durchfallartig ist, muss man sofort zu einem Arzt oder einer Ärztin. (1)<br>**D:** Die Stuhlkonsistenz ändert sich mit dem Wetter. Bei Sonnenschein ist der Stuhl häufig weicher. (4)
**stoolcolor**	**Andrea muss dringend zur Toilette. Als sie ihren Stuhlgang erledigt hat, bemerkt sie, dass ihr Stuhl leicht verfärbt ist. Sie hatte allerdings in der letzten Zeit nie Bauchweh oder andere Beschwerden. Was kann das bedeuten?**<br>**A:** Andrea hat vermutlich nichts Schlimmes. Leichte Verfärbungen können durch bestimmtes Essen auftreten. (4)<br>**B:** Andrea hat bestimmt eine schlimme Krankheit. Der Stuhl verfärbt sich nur, wenn Blut enthalten ist. (3)<br>**C:** Andrea hat vermutlich eine Verstopfung. Verfärbter Stuhl tritt nur bei Verstopfungen auf. (2)<br>**D:** Andrea hat sich die Haare gefärbt. Meistens färbt sich der Stuhl entsprechend der Haarfarbe. (1)
**doctor**	**Was ist bei einem Arztbesuch wegen Bauchschmerzen am wichtigsten?**<br>**A:** Das Gespräch mit dem Arzt oder der Ärztin, um wichtige Informationen über mögliche Ursachen der Bauchschmerzen zu bekommen. (1)<br>**B:** Die Untersuchung mit dem Otoskop, um den Bauch auf einem Bildschirm zu zeigen und Ursachen zu erkennen. (2)<br>**C:** Eine Spritze, um die Schmerzen zu lindern und weiteren Bauchschmerzen vorzubeugen. (4)<br>**D:** Die Untersuchung mit dem Reflexhammer, um zu überprüfen, ob der Bauch ganz normal reagiert. (3)
**medication**	**Leonie hat funktionelle Bauchschmerzen. Wenn sie wieder Bauchschmerzen hat, nimmt sie jedes Mal Schmerztabletten, doch meist wird der Bauchschmerz davon nicht besser. Heute geht sie zum Arzt. Was wird ihr Arzt ihr wohl sagen?**<br>**A:** Bei funktionellen Bauchschmerzen helfen Schmerztabletten in der Regel nicht. (1)<br>**B:** Bei funktionellen Bauchschmerzen muss man jeden Morgen zur Sicherheit eine Schmerztablette nehmen. (3)<br>**C:** Bei funktionellen Bauchschmerzen muss man stärkere Schmerztabletten nehmen, als man sie normalerweise zu Hause hat. (2)<br>**D:** Bei funktionellen Bauchschmerzen helfen keine Schmerztabletten, sondern regelmäßige Spritzen. (4)

**nutrition**	**Marie hat gelernt, dass auch die Ernährung bei funktionellen Bauchschmerzen eine Rolle spielt. Wie sollte man sich am besten ernähren?**<br>**A:** Am besten ist es, sich ganz normal und gesund zu ernähren. (2)<br>**B:** Man sollte kein Gluten, keine Laktose und keine Fruktose essen. (1)<br>**C:** Man sollte viele Kalorien essen, um wieder zuzunehmen. (4)<br>**D:** Am besten helfen Süßigkeiten, weil die über die Bauchschmerzen hinwegtrösten. (3)
**sleep**	**Was sollte man kurz vor dem Schlafengehen bei funktionellen Bauchschmerzen tun?**<br>**A:** An etwas Schönes denken, zum Beispiel an drei Dinge, die an dem Tag gut waren. (3)<br>**B:** Sich überlegen, wie die Bauchschmerzen am nächsten Tag sein könnten, um gut vorbereitet zu sein. (2)<br>**C:** Genau in sich hineinhorchen, ob man gerade wieder Bauchweh hat. (1)<br>**D:** Einen Handstand machen, damit sich die Bauchorgane richtig ordnen. (4)
**answer**	**Bei funktionellen Bauchschmerzen…**<br>**A:** … werden Signale aus dem Magen-Darm-Bereich im Gehirn falsch verstanden. (3)<br>**B:** … funktioniert der Magen-Darm-Bereich nur mit bestimmten Medikamenten vernünftig. (2)<br>**C:** … reagiert der Bauch darauf, wenn in der Nähe im Polizeiauto über Funk gesprochen wird. (1)<br>**D:** … findet der Arzt oder die Ärztin immer eine Entzündung im Bauch. (4)
**limitation**	**Mia hat häufig Bauchschmerzen. Sie kann wegen der Schmerzen oft nicht zur Schule gehen oder an Aktivitäten teilnehmen, die sie gerne mag. Ihre Kinderärztin hat schon viele Untersuchungen gemacht, konnte aber keine Erkrankung feststellen. Was könnte Mia haben?**<br>**A:** Mia könnte funktionelle Bauchschmerzen haben. Dabei hat man häufig Bauchschmerzen, obwohl die Ärztin bei den Untersuchungen nichts findet. (3)<br>**B:** Mia könnte eine Lebensmittelunverträglichkeit haben. Es gibt keine Tests, um diese festzustellen. Deswegen hat ihre Ärztin bisher nichts gefunden. (1)<br>**C:** Mia könnte eine Blinddarmentzündung haben. Diese Krankheit haben Kinder nur sehr selten und sie ist darum schwer im Bauch zu finden. (4)<br>**D:** Mia hat keine Lust zur Schule zu gehen und tut nur so, als ob sie Bauchschmerzen hat. Bei Bauchschmerzen findet man immer eine Erkrankung im Bauch. (2)
**types**	**In welche zwei Arten werden Bauchschmerzen hauptsächlich unterteilt?**<br>**A:** Akute und chronische Bauchschmerzen (1)<br>**B:** Aktuelle und vergangene Bauchschmerzen (3)<br>**C:** Leichte und starke Bauchschmerzen (4)<br>**D:** Angst- und Hunger-Bauchschmerzen (2)
**statement**	**Welche Aussage ist richtig?**<br>**A:** Veränderungen in der Darmbewegung hängen häufig mit Gefühlen zusammen. (1)<br>**B:** Nur 4-mal die Woche Stuhlgang zu haben, ist ungesund. (3)<br>**C:** Bei Darmgeräuschen sollte man sofort einen Arzt oder eine Ärztin aufsuchen. (4)<br>**D:** Spätestens 5 Stunden nach dem Essen werden die Nahrungsreste ausgeschieden. (2)

**gurgle**	**Anna hat gerade gut gefrühstückt. Kurz danach macht sich ihr Bauch bemerkbar und er fängt an, stark zu gluckern. Woher kommt das Gluckern wohl am ehesten**<br>**A:** Der Bauch verarbeitet das Frühstück. Bei der Verdauung entstehen auch gluckernde Geräusche. (4)<br>**B:** Das Frühstück war vermutlich schon schlecht. Weil der Bauch gegen Bakterien ankämpft, entsteht ein Glucker-Geräusch. (3)<br>**C:** Sie hat eine Blinddarmentzündung. Bauchgluckern ist ein wichtiges Alarmsignal. (1)<br>**D:** Sie schreibt in der ersten Schulstunde eine Mathearbeit. Dadurch ist der Bauch „aufgedreht“ und es kommt zu Glucker-Geräuschen. (2)
**stress**	**Lara spielt nach der Schule zwei Mal die Woche Basketball und macht Karate. An den anderen Tagen geht sie zum Trompetenunterricht, wo ein Junge sie immer ärgert. Zu den Hausaufgaben kommt sie meistens erst am späten Abend. Doch sie ist sehr fleißig und möchte Klassenbeste bleiben. Seit einer Weile hat sie Bauchweh und immer wieder Verstopfung oder Durchfall. Was hat Lara wohl?**<br>**A:** Lara hat zu viel Stress. Durch Stress kann die Darmaktivität durcheinandergeraten. (4)<br>**B:** Lara bewegt sich zu wenig. Durch zu wenig Sport arbeitet der Darm zu schnell und es kommt zu Verstopfungen. (1)<br>**C:** Lara hat vermutlich eine Lebensmittelunverträglichkeit. Durch die vielen Aktivitäten konnte sie das noch nicht mit einem Arzt oder einer Ärztin abklären. (3)<br>**D:** Lara hat einen sogenannten „Musikerdarm“. Durch die Bauchanspannung beim Trompete spielen gerät der Darm häufig durcheinander. (2)
**perception**	**Wie nehmen wir Bauchschmerzen wahr?**<br>**A:** Durch Informationen, die der Bauch an das Gehirn sendet. (4)<br>**B:** Durch Bewegungen der Bauchorgane, die durch Schmerzen entstehen. (2)<br>**C:** Durch die Aktivität vom Nervenzentrum im Bauchnabel. (1)<br>**D:** Durch laute Geräusche, die wir aus dem Bauch hören. (3)
**obstipation**	**Anton hat seit Monaten immer wieder Bauchweh. Meistens hat er selten und sehr harten Stuhlgang, ab und zu aber auch wieder sehr flüssigen Stuhlgang. Beim Toilettengang muss er oft feste drücken und häufig tut es dann auch weh. Was hat Anton wohl?**<br>**A:** Anton hat vermutlich eine Verstopfung. Dabei verstopft harter Stuhl den Darm und nur sehr flüssiger Stuhl kann manchmal vorbeifließen. (4)<br>**B:** Anton hat offensichtlich einen Magen-Darm-Infekt. Dabei ist der Stuhlgang zunächst ganz hart und dann flüssig. (3)<br>**C:** Anton hat bestimmt eine schlimme Darmerkrankung, denn normalerweise ist der Stuhlgang immer gleich. (1)<br>**D:** Anton hat vielleicht eine Lebensmittelvergiftung. Abwechselnd harter und flüssiger Stuhl sind eindeutige Anzeichen dafür. (2)

**food poisioning**	**Ben geht es echt schlecht. Er hat gestern Nudeln gegessen, die etwas komisch geschmeckt und gerochen haben. Heute musste er sich schon zweimal übergeben und hat schlimme Bauchkrämpfe. Was ist bei Ben die wahrscheinlichste Erklärung?**<br>**A:** Ben hat wohl eine Lebensmittelvergiftung. Die kann von verdorbenem Essen ausgelöst werden und führt zu Erbrechen. (1)<br>**B:** Ben denkt zu viel an seinen Theaterauftritt in zwei Wochen und hat einen "nervösen" Magen. Mit dem Essen hat das nichts zu tun. (4)<br>**C:** Ben hat eine Laktose-Unverträglichkeit. Offensichtlich verträgt er keine Nudeln und hat deshalb Bauchschmerzen. (2)<br>**D:** Ben hatte sich eigentlich vorgenommen, weniger Kohlenhydrate zu essen. Das schlechte Gewissen zeigt sich durch die Bauchschmerzen. (3)
**influence**	**Welche Bereiche im Leben können funktionelle Bauchschmerzen beeinflussen? Kreuze die Antwort an, die die Frage am vollständigsten beantwortet.**<br>**A:** Alle Antworten stimmen (2)<br>**B:** Schule und Freizeit (3)<br>**C:** Familie und Freunde (4)<br>**D:** Gefühle und Laune (1)
**distraction**	**Was hat Ablenkung mit funktionellen Bauchschmerzen zu tun?**<br>**A:** Ablenkung bewirkt, dass sich das Gehirn auf andere Dinge als den Bauchschmerz konzentriert. (2)<br>**B:** Durch Ablenkung werden bestimmte Stoffe produziert, die die Entzündung im Bauch lindern. (3)<br>**C:** Ablenkung durch Fernsehen oder Computerspielen hilft bei Kindern und Jugendlichen am längsten. (4)<br>**D:** Nichts, durch Ablenkung können die Bauchschmerzen nicht besser werden. (1)
**help**	**Tims Ärztin hat herausgefunden, dass er funktionelle Bauchschmerzen hat. Sollte Tim seinen Eltern von den Bauchschmerzen erzählen?**<br>**A:** Ja, denn sie können ihn unterstützen und dabei helfen, ihn abzulenken. (3)<br>**B:** Ja, dann können sie ihm helfen, indem sie ihm eine Schmerztablette geben. (4)<br>**C:** Nein, damit würde er ihnen nur unnötig Sorgen machen. (2)<br>**D:** Nein, denn wenn es ein Geheimnis bleibt, verschwindet der Schmerz schnell wieder. (1)
**soccer**	**Jan hat funktionelle Bauchschmerzen. Morgen hat er ein wichtiges Fußballspiel und er sollte eigentlich trainieren. Nun sind die Bauchschmerzen wieder da. Was sollte Jan jetzt tun?**<br>**A:** Er sollte für das Fußballspiel trainieren, da Ablenkung am besten gegen den Bauchschmerz hilft. (2)<br>**B:** Er sollte sich hinlegen und ausruhen, da Sport die Bauchschmerzen verschlimmert. (1)<br>**C:** Er sollte das Fußballspiel morgen absagen und sich auf seine Bauchschmerzen konzentrieren, damit sie verschwinden. (4)<br>**D:** Er sollte eine Tafel dunkle Schokolade essen, damit er sich durch die besonderen Inhaltsstoffe schnell besser fühlt. (3)

**warmth**	**Finn hat etwas Interessantes über Wärme bei funktionellen Bauchschmerzen gelesen, aber er kann sich nicht mehr richtig daran erinnern. Was wird Finn über Wärme bei funktionellem Bauchschmerz gelesen haben?**<br>**A:** Wärmflaschen helfen bei funktionellem Bauchschmerz, wenn überhaupt, nur kurz. (1)<br>**B:** Man sollte sich ein Wärmekissen mit heilsamen Körnern vom Arzt oder der Ärztin verschreiben lassen. (2)<br>**C:** Jegliche Art von Wärmflasche oder Wärmekissen helfen bei funktionellen Bauchschmerzen sehr gut. (4)<br>**D:** Wärmflaschen mit Sprudelfunktion zeigen bei funktionellen Bauchschmerzen die beste Wirkung. (3)

[1]Correct response option is shown first (A) here.

[2]First ten items displayed are the final Child A-PKQ items.

[3]The numbering shows the actual order of the response options as they were used in the questionnaire.

Table S2

Table S2. Full A-PKQ Child Version – Non-validated English translation.

	Questions and response options[1]
Abbreviated Items[2]	
**stoolform**	**Which statement about stool shape and consistency is correct?**<br>**A:** The shape of the stool varies from time to time. The sausage shape is the most common. (2)[3]<br>**B:** If the stool is very hard, move as little as possible. It will then become softer and come out more easily. (3)<br>**C:** If your stool becomes really soft or watery, you should see a doctor immediately. (1)<br>**D:** Stool consistency changes with the weather. The stool is often softer in sunny weather. (4)
**stoolcolor**	**Andrea urgently needs to go to the toilet. After she has finished her bowel movement, she notices that her stool is slightly discolored. However, she has not had any stomach pains or other complaints recently. What could this mean?**<br>**A:** Andrea probably has nothing serious. Slight discoloration can occur due to certain foods. (4)<br>**B:** Andrea must have a serious illness. The stool only becomes discolored if it contains blood. (3)<br>**C:** Andrea probably has constipation. Discolored stool only occurs with constipation. (2)<br>**D:** Andrea has recently dyed her hair. Stool is usually the same color as the hair. (1)
**doctor**	**What is most important when visiting a doctor for abdominal pain?**<br>**A:** Having a conversation with the doctor to gather important information about possible causes of abdominal pain. (1)<br>**B:** Undergoing an examination with an otoscope to show the abdomen on a screen and identify potential causes. (2)<br>**C:** Receiving an injection to relieve the pain and prevent further abdominal pain. (4)<br>**D:** Undergoing an examination with a reflex hammer to check whether the abdomen is reacting normally. (3)
**medication**	**Leonie experiences functional abdominal pain. She takes painkillers whenever she has stomach pain, but they don't usually help. Today she goes to see a doctor. What will her doctor likely tell her?**<br>**A:** Painkillers do not usually help with functional abdominal pain. (1)<br>**B:** If you have functional abdominal pain, you should take a painkiller every morning to be on the safe side. (3)<br>**C:** If you have functional abdominal pain, you need to take stronger painkillers than what you may normally have at home. (2)<br>**D:** For functional abdominal pain, regular injections are more effective than painkillers. (4)
**nutrition**	**Marie has learned that diet also plays a role in functional abdominal pain. What is the best way to eat?**<br>**A:** Eating a normal and healthy diet. (2)<br>**B:** You should not eat gluten, lactose, or fructose. (1)<br>**C:** You should eat a lot of calories to gain weight. (4)<br>**D:** Sweets help best because they comfort the stomach ache. (3)

**sleep**	**What should you do just before going to bed if you have functional abdominal pain?**<br>**A:** Think of something nice. For example, three good things that happened that day. (3)<br>**B:** Think about what your stomach ache might be like the next day, so that you are prepared. (2)<br>**C:** Pay careful attention to your body for signs of a stomach ache starting. (1)<br>**D:** Do a handstand so that the abdominal organs are properly organized. (4)
**answer**	**In the case of functional abdominal pain…**<br>**A:** … signals from the gastrointestinal area are misinterpreted in the brain. (3)<br>**B:** … the gastrointestinal tract only functions properly with certain medications. (2)<br>**C:** … the abdomen reacts when people are speaking on the radio in the police car nearby. (1)<br>**D:** … the doctor will always find inflammation in the abdomen. (4)
**limitation**	**Mia often has stomach pains. She often can't go to school or take part in activities she likes because of the pain. Her pediatrician has already done many tests, but has been unable to diagnose Mia with any illness. What could Mia have?**<br>**A:** Mia could have functional abdominal pain. Abdominal pain can occur even though the doctor doesn't find anything wrong during examinations. (3)<br>**B:** Mia could have a food intolerance. There are no tests to determine this, which is why her doctor hasn't found anything yet. (1)<br>**C:** Mia could have appendicitis. This disease is very rare in children and is difficult to find in the abdomen. (4)<br>**D:** Mia doesn't feel like going to school and is only pretending to have a stomach ache. Stomach aches are only caused by an illness in your stomach. (2)
**types**	**What are the two main types of abdominal pain?**<br>**A:** Acute and chronic abdominal pain (1)<br>**B:** Current and past abdominal pain (3)<br>**C:** Mild and severe abdominal pain (4)<br>**D:** Anxiety and hunger-related abdominal pain (2)
**statement**	**Which statement is correct?**<br>**A:** Changes in bowel movements are often linked to emotions. (1)<br>**B:** Having a bowel movement only 4 times a week is unhealthy. (3)<br>**C:** If you have bowel sounds, you should consult a doctor immediately. (4)<br>**D:** Food residues are usually excreted no later than 5 hours after eating. (2)
**gurgle**	**Anna has just finished a hearty breakfast. Shortly afterwards, her stomach makes itself known and begins to gurgle loudly. Where is the gurgling most likely coming from?**<br>**A:** The stomach is processing breakfast. Gurgling noises are produced during digestion. (4)<br>**B:** Her breakfast was probably contaminated. The stomach is fighting bacteria, which causes the gurgling noise. (3)<br>**C:** She has appendicitis. Abdominal gurgling signals a medical emergency. (1)<br>**D:** She is feeling anxious about writing a math test during the first lesson at school. The thought of this "turns her stomach" and it makes gurgling noises. (2)

**stress**	**Lara plays basketball and does karate twice a week after school. On the other days, she goes to trumpet lessons, where a boy always annoys her. She usually doesn't get to her homework until late in the evening. But she is very hard-working and wants to stay at the top of her class. She has had a stomach ache for a while now and is always constipated or has diarrhea. What do you think is wrong with Lara?**<br>**A:** Lara has too much stress. Stress can disrupt intestinal activity. (4)<br>**B:** Lara does too little exercise. Too little exercise causes the bowel to work too quickly and leads to constipation. (1)<br>**C:** Lara probably has a food intolerance. Due to her many activities, she has not yet been able to clarify this with a doctor. (3)<br>**D:** Lara has what is known as a "musician's bowel". The abdomen tenses when playing the trumpet, which makes the bowel get mixed up. (2)
**perception**	**How do we perceive abdominal pain?**<br>**A:** Through information that the stomach sends to the brain. (4)<br>**B:** Through movements of the abdominal organs caused by pain. (2)<br>**C:** Through the activity of the nerve center located in the navel. (1)<br>**D:** Through loud noises coming from our stomach. (3)
**obstipation**	**Anton has had stomach pains for several months. Most of the time he has infrequent and very hard bowel movements, but sometimes he has very loose bowel movements. He often has to push hard when using the toilet, which hurts. What do you think Anton has?**<br>**A:** Anton probably has constipation. Hard stools block the bowel, so and only loose stools can pass through. (4)<br>**B:** Anton obviously has a gastrointestinal infection. His stools are very hard at first and then they become loose. (3)<br>**C:** Anton must have a serious bowel disease, because normally his bowel movements look the same. (1)<br>**D:** Anton may have food poisoning. Alternating hard and liquid stools are clear signs of this. (2)
**food poisoning**	**Ben is feeling really unwell. Yesterday, he ate pasta that tasted and smelled a bit strange. Today he has already thrown up twice and has bad stomach cramps. What is the most likely explanation for Ben's symptoms?**<br>**A:** Ben probably has food poisoning. This can be caused by eating spoiled food, which leads to vomiting. (1)<br>**B:** Ben is thinking too much about his theater performance happening in two weeks and has a "nervous" stomach. It has nothing to do with the food. (4)<br>**C:** Ben has lactose intolerance. He obviously can't tolerate pasta and therefore has a stomach ache. (2)<br>**D:** Ben had originally planned to eat less carbohydrates. His guilt is causing in his stomach ache. (3)
**influence**	**Which areas of your life can be affected by functional abdominal pain? Select the best answer.**<br>**A:** All answers are correct (2)<br>**B:** School and leisure time (3)<br>**C:** Relationships with family and friends (4)<br>**D:** Feelings and mood (1)

**distraction**	**How does distraction affect functional abdominal pain?**<br>**A:** Distraction makes the brain focus on things other than the abdominal pain. (2)<br>**B:** Distraction produces substances that relieve inflammation in the abdomen. (3)<br>**C:** Watching TV or playing computer games helps distract children and young people for the longest amount of time. (4)<br>**D:** It doesn't. Distraction can't help make a stomach ache better. (1)
**help**	**Tim's doctor has discovered that he has functional abdominal pain. Should Tim tell his parents about the abdominal pain?**<br>**A:** Yes, because they can support him and help to distract him. (3)<br>**B:** Yes, then they can help him by giving him a painkiller. (4)<br>**C:** No, he would only worry them unnecessarily. (2)<br>**D:** No, because if it remains a secret, the pain will quickly disappear. (1)
**soccer**	**Jan has functional abdominal pain. He has an important soccer match tomorrow that he must train for, but his abdominal pain has returned. What should Jan do?**<br>**A:** He should train for the soccer game, as distraction is the best way to lessen abdominal pain. (2)<br>**B:** He should lie down and rest, as exercise aggravates abdominal pain. (1)<br>**C:** He should cancel the soccer game tomorrow and concentrate on his stomach pains to make them go away. (4)<br>**D:** He should eat a bar of dark chocolate, it has special ingredients that will make him feel better quickly. (3)
**warmth**	**Finn read something interesting about using heat to treat functional abdominal pain, but he can't quite remember it. What might Finn have read about using heat for functional abdominal pain?**<br>**A:** Hot water bottles only provide short-term relief, if any, from functional abdominal pain. (1)<br>**B:** Your doctor will prescribe a heating pad with healing grains inside of it. (2)<br>**C:** Any type of hot water bottle or heating pad can help relieve functional abdominal pain. (4)<br>**D:** Hot water bottles with a bubble function are the most effective for treating functional abdominal pain. (3)

[1]Correct response option is shown first (A) here.
[2]First ten items displayed are the final Child A-PKQ items.
[3]The numbering shows the actual order of the response options as they were used in the questionnaire.

Table S3

Table S3. Full A-PKQ Parent Version – Original German Version.

	**Questions and response options**[1]
Abbreviated Items[2]	
**children ap**	**Was sollte man vor allem bei kleineren Kindern mit Bauchschmerz bedenken?**<br>**A:** Kleinere Kinder interpretieren jegliche Signale aus dem Bauch schnell als Schmerz. Daher sollte man zuerst nach alltäglichen Gründen (z.B. Hunger) fragen. (4)[3]<br>**B:** Bei kleineren Kindern bedeutet Bauchschmerz immer etwas sehr Kritisches. Bei ersten Anzeichen sollte sofort ein Arzt aufgesucht werden. (3)<br>**C:** Vor allem jüngeren Kindern fällt es schwer ihre Gefühle mitzuteilen. Daher sollte man sie regelmäßig nach Schmerzen befragen. (2)<br>**D:** Jüngere Kinder möchten noch besonders viel Aufmerksamkeit bekommen. Bauchschmerz ist daher immer ein Vorwand. (1)
**move**	**Welche Art der Bewegung hilft bei funktionellen Bauchschmerzen besonders gut?**<br>**A:** Die Art der Bewegung ist unwichtig, solange sie regelmäßig stattfindet und Spaß macht. (4)<br>**B:** Ballsportarten sind besonders gut, da sie im Team stattfinden. (3)<br>**C:** Turnen ist am besten geeignet, da der Körper dabei ausgewogen belastet wird. (2)<br>**D:** Bei funktionellem Bauchschmerz sollte man möglichst vorsichtig Sport betreiben, z.B. langsam Spazieren gehen. (1)
**fap daily**	**Anton leidet an funktionellen Bauchschmerzen. Schon morgens fangen die Bauchschmerzen an und er möchte wegen der Schmerzen nicht zur Schule gehen. Was raten Sie Antons Eltern?**<br>**A:** Anton ermutigen in die Schule zu gehen, da ein normaler Alltag funktionelle Bauchschmerzen mittelfristig verbessert. (1)<br>**B:** Anton erlauben im Bett zu bleiben, damit er sich ausruhen kann, bis die Bauchschmerzen verschwinden. (4)<br>**C:** Anton zum Aufstehen überreden und ihn den Tag mit einer Wärmflasche auf der Couch verbringen lassen. (3)<br>**D:** Mit Anton zum Arzt fahren und eine entsprechende Medikation verschreiben lassen. (2)
**microbiome**	**Was ist das Mikrobiom?**<br>**A:** Mikroorganismen, die vor allem den Darm besiedeln und vermutlich funktionellen Bauchschmerz mitbeeinflussen. (1)<br>**B:** Ein homöopathischer Mythos, für den es bisher keinerlei Nachweise gibt. (2)<br>**C:** Eine Ansammlung von Viren und Hefezellen, die das Immunsystem gegen funktionellen Bauchschmerz aktivieren. (4)<br>**D:** Eine Art innere Neurodermitis, die Entzündungen verursacht und zu funktionellem Bauchschmerz führt. (3)

**change fap**	**Wie verändern sich funktionelle Bauchschmerzen, wenn man besonders auf seinen Bauch achtet?**<br>**A:** Die Schmerzen nehmen zu, da das Gehirn noch mehr Signale aus dem Bauch wahrnimmt und diese fälschlicherweise als Schmerz deutet. (2)<br>**B:** Mehr oder weniger Aufmerksamkeit auf den Bauch macht keinen Unterschied bei funktionellen Bauchschmerzen. (3)<br>**C:** Die Schmerzen werden weniger, da der Körper die Signale besser wahrnimmt und darauf reagieren kann. (1)<br>**D:** Aufmerksamkeit auf funktionellen Bauchschmerz fördert die Durchblutung und trägt dadurch zur geringeren Schmerzwahrnehmung bei. (4)
**occurrence**	**Valerie, die Tochter eines Freundes hat häufiger Bauchschmerzen. Manchmal treten sie einfach so auf, andere Male nach dem Verzehr von Fruktose. Was sollte Ihr Freund am besten tun?**<br>**A:** Einen Arzt oder eine Ärztin aufsuchen, um gemeinsam mögliche Ursachen und Vorgehensweisen abzuklären. (3)<br>**B:** Valerie für einige Monate nur fruktosefreie Nahrung geben, um zu prüfen, ob die Bauchschmerzen weniger werden. (4)<br>**C:** Nichts, da häufigere Bauchschmerzen bei Kindern normal sind und nach der Pubertät wieder verschwinden. (2)<br>**D:** Vor allem stark fruktosehaltige Nahrungsmittel verabreichen, damit sich der Darm des Kindes daran gewöhnt. (1)
**help fap**	**Was hilft bei funktionellen Bauchschmerzen am besten?**<br>**A:** Ablenkung, damit das Gehirn andere Dinge als die Signale aus dem Bauch fokussiert. (1)<br>**B:** Medikamente, die schmerzlindernd wirken und den Entstehungsherd bekämpfen. (4)<br>**C:** Nahrungsumstellungen, die den Magen-Darm-Bereich wieder ins Gleichgewicht bringen. (3)<br>**D:** Bettruhe, damit sich der Körper ausruhen und erholen kann. (2)
**intolerance**	**Bei einer Fruktose- und Laktoseunverträglichkeit…**<br>**A:** … fehlen dem Körper Enzyme oder Proteine zur Aufnahme der Nahrungsbestandteile. (4)<br>**B:** … reagiert der Körper mit einer Immunantwort, wie bei einer Allergie. (3)<br>**C:** … sind die entsprechenden Transporter in einer zu hohen Konzentration im Blut zu finden. (1)<br>**D:** … ist immer eine vorherige Darmerkrankung der Auslöser. (2)
**fap**	**Was sind funktionelle Bauchschmerzen?**<br>**A:** Bei funktionellem Bauchschmerz ist die Kommunikation der Darm-Hirn Achse gestört. (1)<br>**B:** Durch Entzündungen an bestimmten Hirnarealen entstehen Bauchschmerz-Signale. (4)<br>**C:** Strukturelle Veränderungen im enterischen Nervensystem unterbinden jegliche Signalübermittlung zum Gehirn, wodurch ein Dauerschmerz entsteht. (3)<br>**D:** Die Darmabläufe funktionieren nicht richtig und die Nahrung kann nicht angemessen verarbeitet werden. (2)

**recurrent**	**Was haben ein Magen-Darm-Infekt, eine Verstopfung und eine Magenschleimhautentzündung gemeinsam?**<br>**A:** Sie können sich nach der akuten Erkrankung zu wiederkehrenden Bauchschmerzen entwickeln. (2)<br>**B:** Es sind akute Erkrankungen, die in jedem Fall nach kurzer Zeit vollständig verschwinden. (3)<br>**C:** Bei allen drei Erkrankungen kommt der Stuhlgang sehr selten, kann jedoch auch durchfallartig auftreten. (4)<br>**D:** Alle Erkrankungen werden in etwa 90% der Fälle durch Helicobacter Bakterien hervorgerufen. (1)
**relaxation**	**Wie sollte man bei funktionellen Bauchschmerzen mit Ruhe und Entspannung umgehen?**<br>**A:** Es sollten Ruhe oder Entspannungspausen bei funktionellen Bauchschmerzen geplant werden, doch Aktivität ist genauso wichtig. (2)<br>**B:** Bei funktionellen Bauchschmerzen sollte man keine Pausen machen, da Aktivität das beste Hilfsmittel ist. (3)<br>**C:** Nur Yoga und Pilates sind bei funktionellem Bauchschmerz sinnvoll, da allein diese Entspannungsmethoden gute Ergebnisse zeigen. (1)<br>**D:** Die meiste Zeit des Tages sollte ruhig ablaufen und man sollte nur etwa eine Stunde aktiv sein. (4)
**ap origin**	**Marco, der Sohn einer Freundin, hat regelmäßig Bauchschmerzen. Meistens beginnen sie kurz vor der Schule und sind wieder verschwunden, sobald Marco wieder zu Hause ist. Ihre Freundin war mit Marco schon mehrfach beim Arzt, doch nie wurde eine Erkrankung gefunden. Was könnte die Bauchschmerzen am wahrscheinlichsten verursachen?**<br>**A:** Es könnte sein, dass Marco einen hohen Leistungsdruck empfindet oder es ihm in der Schule nicht gut geht. Diese negativen Gefühle und Stress führen häufig zu funktionellem Bauchschmerz. (4)<br>**B:** Viele Krankheiten, die Bauchschmerzen verursachen, sind sehr schwer nachzuweisen. Die wahrscheinlichste Erklärung ist, dass Marco eine seltene Erkrankung hat, die bisher kein Spezialist erkennen konnte. (2)<br>**C:** Viele Kinder sind eher Nachteulen und es fällt ihnen schwer morgens in den Tag zu starten. Das ist ganz normal. Ab nachmittags sind sie leistungsfähiger, daher verschwinden die Bauchschmerzen um diese Zeit wieder. (1)<br>**D:** Marco hat vermutlich keine Lust zur Schule zu gehen und täuscht die Bauchschmerzen vor. So erhofft er sich Mitleid und vom Schulbesuch befreit zu werden. (3)
**nutrition rules**	**Welche Ernährungsregeln sollte man bei funktionellem Bauchschmerz beachten?**<br>**A:** Man sollte sich gesund und ausgewogen ernähren. (3)<br>**B:** Der Verzehr von Hülsenfrüchten sollte gesteigert werden. (2)<br>**C:** Es sollte ein spezieller Ernährungsplan mit vielen Ballaststoffen erstellt werden. (1)<br>**D:** Der tägliche Konsum von Kohlenhydraten sollte reduziert werden. (4)
**fap influence**	**Kreuzen Sie an, welche der vier Antwortmöglichkeiten ausschließlich Einflussfaktoren für funktionellen Bauchschmerz zeigt.**<br>**A:** Stress, Gefühle, Mikrobiom und Aufmerksamkeit. (3)<br>**B:** Aufmerksamkeit, Gefühle, Wetterwechsel und Darmflora. (1)<br>**C:** Krafttraining, Darmflora, Stress und Gefühle. (2)<br>**D:** Aufmerksamkeit, Krafttraining, Stress und Wetterwechsel. (4)

**morbus colitis**	**Was sind Morbus Crohn und Colitis Ulcerosa?**<br>**A:** Chronisch entzündliche Darmerkrankungen, die schubweise auftreten und meist mit Medikamenten behandelt werden müssen. (4)<br>**B:** Akute Darmerkrankungen, die in ihren Ausprägungen fast identisch sind und meist von jedem Kind einmal durchlebt werden. (1)<br>**C:** Entzündungen, die für etwa ein Jahr lang wiederkehrend auftreten und dann vollständig verschwinden. (4)<br>**D:** Zwei funktionelle Bauchschmerzarten, die vor allem im Jugendalter durch Stress verursacht werden. (2)
**gastro pain**	**Der Sohn Ihrer Freundin Clara hat einen Magen-Darm-Infekt. Da Sie selbst mit Ihren Kindern schon häufiger solche Infekte durchlebt haben, fragt Clara Sie um Rat. Was sollten Sie Clara raten?**<br>**A:** „Er sollte sich viel ausruhen. Achte auch darauf, dass er genug Flüssigkeit und Elektrolyte zu sich nimmt." (1)<br>**B:** „Fahr sofort ins Krankenhaus. Nur dort bekommt ihr eine angemessene Behandlung." (4)<br>**C:** „Beobachte seinen Stuhlgang. Sobald er Durchfall bekommt, solltet ihr dringend einen Arzt aufsuchen." (3)<br>**D:** „Geh mit ihm zum Gastroenterologen oder zur Gastroenterologin. Es sollte sofort abgeklärt werden, ob sich daraus ein Reizdarm entwickeln wird." (2)
**ap development**	**Wodurch entstehen Bauchschmerzen?**<br>**A:** Bauchschmerzen können viele Ursachen haben und durch Störungen in der Funktion oder Struktur des Magen-Darm-Bereichs hervorgerufen werden. (3)<br>**B:** Bauchschmerzen entstehen immer aus organischen Gründen, wie zum Beispiel einer Unverträglichkeit oder Entzündung im Magen-Darm-Bereich. (2)<br>**C:** Bauchschmerzen entstehen im Gehirn und werden daher in fast allen Fällen durch emotionale Belastung hervorgerufen. (4)<br>**D:** Bauchschmerzen sind in den meisten Fällen ein Vorwand, um Aufmerksamkeit und Zuwendung zu erhalten. (1)
**irritable bowel**	**In einer Quiz-Show kommt eine Frage zu funktionellem Bauchschmerz. In der Frage geht es darum, ob funktioneller Bauchschmerz und Reizdarm genau dasselbe sind. Welche Antwort stimmt?**<br>**A:** „Nein, Reizdarm ist eine von mehreren Unterarten des funktionellen Bauchschmerzes." (1)<br>**B:** „Nicht ganz, der Reizdarm äußert sich zusätzlich durch eine Magenschleimhautentzündung." (3)<br>**C:** „Fast, für funktionellen Bauchschmerz müssen drei weitere Diagnosekriterien erfüllt sein." (4)<br>**D:** „Ja, die beiden Begriffe sind Synonyme und bezeichnen dieselbe Erkrankung und die gleichen Symptome." (2)

[1]Correct response option is shown first (A) here.
[2]First ten items displayed are the final Parent A-PKQ items.
[3]The numbering shows the actual order of the response options as they were used in the questionnaire.

Table S4

Table S4. Full A-PKQ Parent Version – Non-validated English translation.

	Questions and response options[1]
Abbreviated Items[2]	
**children ap**	**What should be considered, regarding younger children with abdominal pain?**<br>**A:** Younger children often interpret any signals coming from the stomach as pain. Therefore, you should first ask them about everyday reasons (e.g. hunger). (4)[3]<br>**B:** In younger children, abdominal pain always indicates something very serious. At the first sign of pain, a doctor should be consulted immediately. (3)<br>**C:** Younger children in particular find it difficult to communicate their feelings. You should therefore regularly ask them about pain. (2)<br>**D:** Younger children often seek attention. A tummy ache is always a pretext. (1)
**move**	**What type of exercise particularly helps with functional abdominal pain?**<br>**A:** The type of exercise isn't important as long as it is done regularly and is fun. (4)<br>**B:** Ball sports are particularly beneficial because they involve teams. (3)<br>**C:** Gymnastics is the most suitable as it puts balanced strain on the body. (2)<br>**D:** If you have functional abdominal pain, you should exercise as carefully as possible, such as going for a slow walk. (1)
**fap daily**	**Anton suffers from functional abdominal pain. The abdominal pain starts in the morning, and he doesn't want to go to school because of it. What advice would you give Anton's parents?**<br>**A:** Encourage Anton to go to school, as sticking to a normal daily routine can improve functional abdominal pain over time. (1)<br>**B:** Allow Anton to stay in bed so that he can rest until the stomach pains disappear. (4)<br>**C:** Convince Anton to get up and let him spend the day on the couch with a hot water bottle. (3)<br>**D:** Take Anton to the doctor to get the appropriate medication prescribed. (2)
**microbiome**	**What is the microbiome?**<br>**A:** Microorganisms that primarily colonize the intestine and are suspected to contribute to functional abdominal pain. (1)<br>**B:** A homeopathic myth for which there is no evidence to date. (2)<br>**C:** A collection of viruses and yeast cells that activate the immune system against functional abdominal pain. (4)<br>**D:** A type of internal neurodermatitis that causes inflammation and leads to functional abdominal pain. (3)
**change fap**	**How does functional abdominal pain change when you pay special attention to your stomach?**<br>**A:** The pain increases because the brain perceives there to be more signals coming from the abdomen, misinterpreting them as pain. (2)<br>**B:** Paying more or less attention to the abdomen doesn't affect functional abdominal pain. (3)<br>**C:** The pain decreases as the body processes signals more effectively and can react to them. (1)<br>**D:** Attending to functional abdominal pain promotes blood circulation and thus contributes to reducing the perception of pain. (4)

**occurrence**	**Valerie, the daughter of a friend, has frequent stomach pains. Sometimes they occur out of the blue, other times they happen after consumating fructose. What should your friend do?**<br>**A:** Consult a doctor to investigate possible causes and discuss next steps together. (3)<br>**B:** Give Valerie only fructose-free food for a few months to see if the abdominal pain diminishes. (4)<br>**C:** Nothing, frequent abdominal pain is common in children, and it typically resolves after puberty. (2)<br>**D:** Provide foods with high fructose content to help the child's intestines become accustomed to them. (1)
**help fap**	**What helps best with managing functional abdominal pain?**<br>**A:** Distraction, which redirects the brain's focus to things other than signals from the gut. (1)<br>**B:** Medication that alleviates pain and combats its source. (4)<br>**C:** Dietary changes that rebalance the gastrointestinal tract. (3)<br>**D:** Staying in bed so that the body can rest and recover. (2)
**intolerance**	**In the case of fructose and lactose intolerance,...**<br>**A:** … the body lacks enzymes or proteins needed to digest these food components. (4)<br>**B:** … the body reacts with an immune response, as if it is responding to an allergy. (3)<br>**C:** … there is an excessive concentration of corresponding transporters in the blood. (1)<br>**D:** … a previous intestinal disease is always the trigger. (2)
**fap**	**What characterizes functional abdominal pain?**<br>**A:** It stems from disrupted communication along the gut-brain axis. (1)<br>**B:** Inflammation in certain areas of the brain generate abdominal pain signals. (4)<br>**C:** Structural changes in the enteric nervous system prevent signal transmission to the brain, resulting in persistent pain. (3)<br>**D:** Impaired intestinal processes lead to improper food processing. (2)
**recurrent**	**What do a gastrointestinal infection, constipation, and gastritis have in common?**<br>**A:** They can develop into recurrent abdominal pain following the acute illness. (2)<br>**B:** These are acute illnesses that typically resolve completely after a short time. (3)<br>**C:** All three diseases feature infrequent bowel movements that can also be diarrhea-like. (4)<br>**D:** In about 90% of cases, these diseases are caused by Helicobacter bacteria. (1)
**relaxation**	**How should you approach managing functional abdominal pain with rest and relaxation?**<br>**A:** Plan periods of rest or relaxation breaks, but activity is just as important. (2)<br>**B:** With functional abdominal pain, you should not take breaks; activity is the best aid. (3)<br>**C:** Only Yoga and Pilates are useful relaxation methods for managing functional abdominal pain. (1)<br>**D:** Most of the day should be spent quietly, and you should be active for only about an hour. (4)

**ap origin**	**Marco, the son of a friend, regularly has stomach aches. They usually start just before school and disappear again as soon as Marco is back home. Your friend has taken Marco to the doctor several times, but no illness has ever been found. What could most likely be causing the stomach pains?**<br>**A:** Marco might feel a lot of pressure to perform or is struggling academically. These negative feelings and stress often manifest as functional abdominal pain. (4)<br>**B:** Many diseases that cause abdominal pain are very difficult to detect. The most likely explanation is that Marco has a rare disease that no specialist has been able to detect. (2)<br>**C:** Many children are night owls who find it difficult to start the day early. This is quite normal. They are more productive in the afternoon, so their stomach ache disappears around this time. (1)<br>**D:** Marco doesn't feel like going to school so he is pretending to have a stomach ache. He hopes to get sympathy and be exempted from going to school. (3)
**nutrition rules**	**What dietary guidelines should be followed for functional abdominal pain?**<br>**A:** Eat a healthy and balanced diet. (3)<br>**B:** Increase your consumption of legumes. (2)<br>**C:** Eat a high-fiber special diet. (1)<br>**D:** Reduce daily carbohydrate intake. (4)
**fap influence**	**Select the option that exclusively lists factors that influence functional abdominal pain.**<br>**A:** Stress, emotions, microbiome, and attention. (3)<br>**B:** Attention, emotions, weather changes, and intestinal flora. (1)<br>**C:** Strength training, intestinal flora, stress, and emotions. (2)<br>**D:** Attention, strength training, stress, and weather changes. (4)
**morbus colitis**	**What are Crohn's disease and Ulcerative colitis?**<br>**A:** Chronic inflammatory bowel diseases that occur in phases and are typically treated with medication. (4)<br>**B:** Acute intestinal diseases, which are almost identical in their manifestations and are usually experienced by every child at least once. (1)<br>**C:** Inflammations that recur for about a year and then disappear completely. (4)<br>**D:** Two types of functional abdominal pain caused by stress, particularly in adolescence. (2)
**gastro pain**	**Your friend Clara's son has a gastrointestinal infection. As you and your children have had such infections, Clara asks you for advice. What advice should you give Clara?**<br>**A:** "He should rest a lot. Also make sure he gets enough fluids and electrolytes. "(1)<br>**B:** "Go to hospital immediately. That is the only place where you will receive appropriate treatment. "(4)<br>**C:** "Observe his bowel movements. If diarrhea develops, you should see a doctor immediately. "(3)<br>**D:** "Take him to a gastroenterologist. It should be clarified immediately whether this will develop into irritable bowel syndrome. "(2)
**ap development**	**What causes abdominal pain?**<br>**A:** Abdominal pain can have many causes, including disorders in the function or structure of the gastrointestinal tract. (3)<br>**B:** Abdominal pain always arises for organic reasons, such as an intolerance or inflammation in the gastrointestinal tract. (2)<br>**C:** Abdominal pain originates in the brain and is therefore almost always caused by emotional stress. (4)<br>**D:** In most cases, stomach pains are an excuse to get attention and care. (1)

**irritable bowel**	**In a quiz show, there is a question about functional abdominal pain – specifically, whether it is identical to irritable bowel syndrome (IBS). Which answer is correct?**<br>**A:** **“**No, irritable bowel syndrome is one of several subtypes of functional abdominal pain. “(1)<br>**B:** “Not quite, irritable bowel syndrome also presents as inflammation of the stomach lining. “(3)<br>**C:** “Almost, for functional abdominal pain, three additional diagnostic criteria must be met. “(4)<br>**D:** **“**Yes, the two terms are synonymous, referring to the same disease and symptoms. “(2)

[1]Correct response option is shown first (A) here.

[2]First ten items displayed are the final Parent A-PKQ items.

[3]The numbering shows the actual order of the response options as they were used in the questionnaire.

Table S5

Table S5. Wald test results for the final item fit by comparing item difficulties by gender and age.

	Gender		Age	
	z-statistic	*p*-value	*z*-statistic	*p*-value
**Gastro-intestinal tract**				
**stoolform**	-0.41	.679	-0.68	.496
**stoolcolor**	-1.31	.189	0.33	.741
**statement**	0.42	.675	-1.13	.258
**Abdominal Pain**				
**doctor**	0.17	.862	0.64	.522
**types**	1.58	.114	-1.80	.073
**Functional Abdominal Pain**				
**answer**	0.34	.735	0.11	.910
**limitation**	-1.38	.169	1.08	.279
**medication**	-0.15	.883	-0.81	.419
**Abdominal Pain Management**				
**nutrition**	0.37	.714	1.46	.143
**sleep**	0.80	.426	0.72	.473

Negative values indicate that the item is easier for girls compared to boys, or for older compared to younger patients.

Table S6

Table S6. Mean-square fit statistics for the final ten items of the A-PKQ child version.

	Outfit *(MSQ)*	Infit *(MSQ)*		Outfit *(MSQ)*	Infit (MSQ)
**Gastro-intestinal tract**			**Functional Abdominal Pain**		
stoolform	0.87	0.85	answer	0.83	0.91
stoolcolor	0.76	0.93	limitation	1.13	1.08
statement	1.11	1.12	medication	0.79	0.87
**Abdominal Pain**			**Abdominal Pain Management**		
doctor	1.30	1.14	nutrition	0.71	0.87
types	0.88	0.93	sleep	0.81	0.93

Table S7

Table S7. Wald test results for item fit by comparing parent item difficulties by age and chronic pain.

	**Age**		**Chronic Pain**	
	z-statistic	*p*-value	*z*-statistic	*p*-value
**Abdominal Pain**				
**ap development**	1.16	.244	-0.39	.695
**children ap**	-0.01	.991	-1.10	.272
**gastro pain**	1.10	.272	0.02	.983
**recurrent**	0.10	.919	-1.21	.228
**intolerance**	0.29	.770	0.02	.984
**morbus colitis**	-0.55	.582	-0.41	.679
**Functional Abdominal Pain**				
**fap**	-0.51	.608	0.98	.327
**irritable bowel**	-0.48	.628	-1.84	.066
**fap influence**	1.18	.239	0.03	.974
**microbiome**	-0.99	.322	0.33	.745
**ap origin**	-0.89	.375	0.45	.652
**change fap**	-0.11	.914	-1.10	.271
**Abdominal Pain Management**				
**nutrition rules**	0.42	.678	1.22	.224
**occurrence**	-1.04	.298	1.78	.075
**fap daily**	0.94	.346	-0.34	.736
**help fap**	0.38	.706	1.20	.228
**move**	-0.83	.405	0.48	.635
**relaxation**	-0.18	.860	-0.12	.902

Negative values indicate that the item is easier for older than younger parents, and easier for parents with chronic pain than for parents without chronic pain.

Table S8

Table S8. Wald test results for final item fit by comparing parent item difficulties by age and chronic pain.

	Age		Chronic Pain	
	z-statistic	*p*-value	*z*-statistic	*p*-value
**Abdominal Pain**				
**children ap**	0.25	.801	-1.47	.141
**recurrent**	0.47	.638	-1.28	.199
**intolerance**	0.51	.609	0.05	.957
**Functional Abdominal Pain**				
**fap**	-0.24	.814	0.99	.321
**microbiome**	-0.93	.355	0.38	.702
**change fap**	0.06	.951	-1.07	.283
**Abdominal Pain Management**				
**occurrence**	-0.86	.390	1.83	.067
**fap daily**	1.03	.303	-0.31	.753
**help fap**	0.60	.547	1.25	.211
**move**	-0.78	.435	0.49	.622

Negative values indicate that the item is easier for older than younger parents, and easier for parents with chronic pain than for parents without chronic pain.

Table S9

Table S9. Mean-square fit statistics for the final 10 items of the A-PKQ parent version.

	Outfit *(MSQ)*	Infit *(MSQ)*
**Abdominal Pain**		
**children ap**	1.32	0.91
**recurrent**	1.21	1.19
**intolerance**	1.21	1.03
**Functional Abdominal Pain**		
**fap**	0.87	0.91
**microbiome**	0.98	1.10
**change fap**	0.73	0.80
**Functional Abdominal Pain Management**		
**occurrence**	1.23	1.19
**fap daily**	0.63	0.82
**help fap**	0.74	0.82
**move**	0.76	0.90

Figure S1

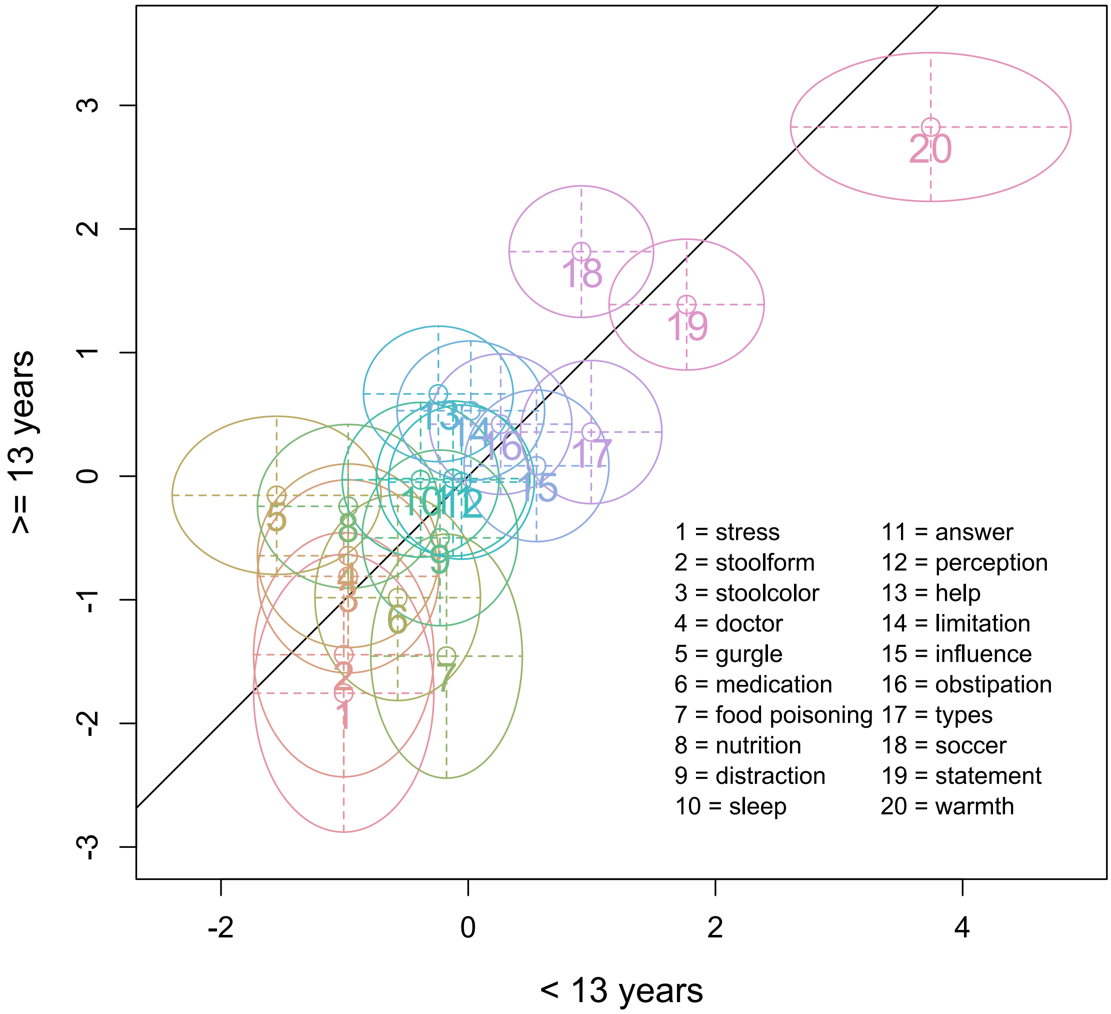

Figure S1. Visualization of Andersen's Likelihood-Ratio Test (LRT) with the child sample split by age. The x-axis displays item parameters for the younger age group (< 13 years); the y-axis displays item parameters for the older age group (>= 13 years). Zero indicates average difficulty, easier items below zero, and more difficult items above zero. The diagonal indicates parameter equality for both age groups. Circles around items show confidence intervals as circles (CI). CI's overlapping with the diagonal indicate well-fitting items, and thus high probability of equality of item parameters across groups. For example, item 5 ('gurgle') is significantly easier for younger than older patients.

Figure S2

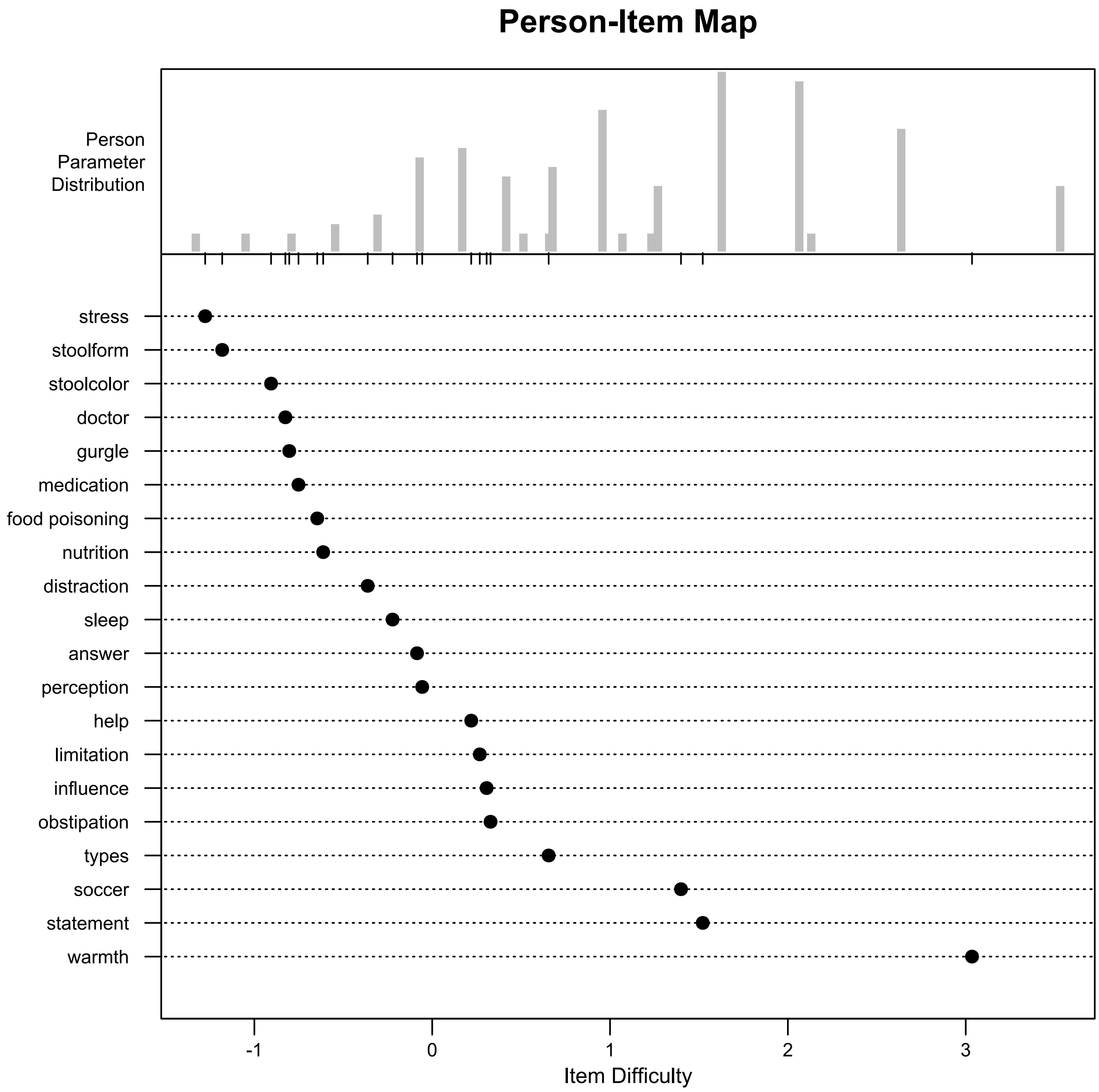

Figure S2. Person-Item map of the A-PKQ child version. The x-axis denotes item difficulty and is adjusted to the range of individual abilities (-1 to 3). Items are listed on the y-axis. The upper panel indicates the distribution of person parameters along the latent dimension. Negative values in the x-axis represent easier items and positive values represent more difficult items. Items around 0 on the x-axis are likely to be solved correctly by patients of average ability.

Figure S3

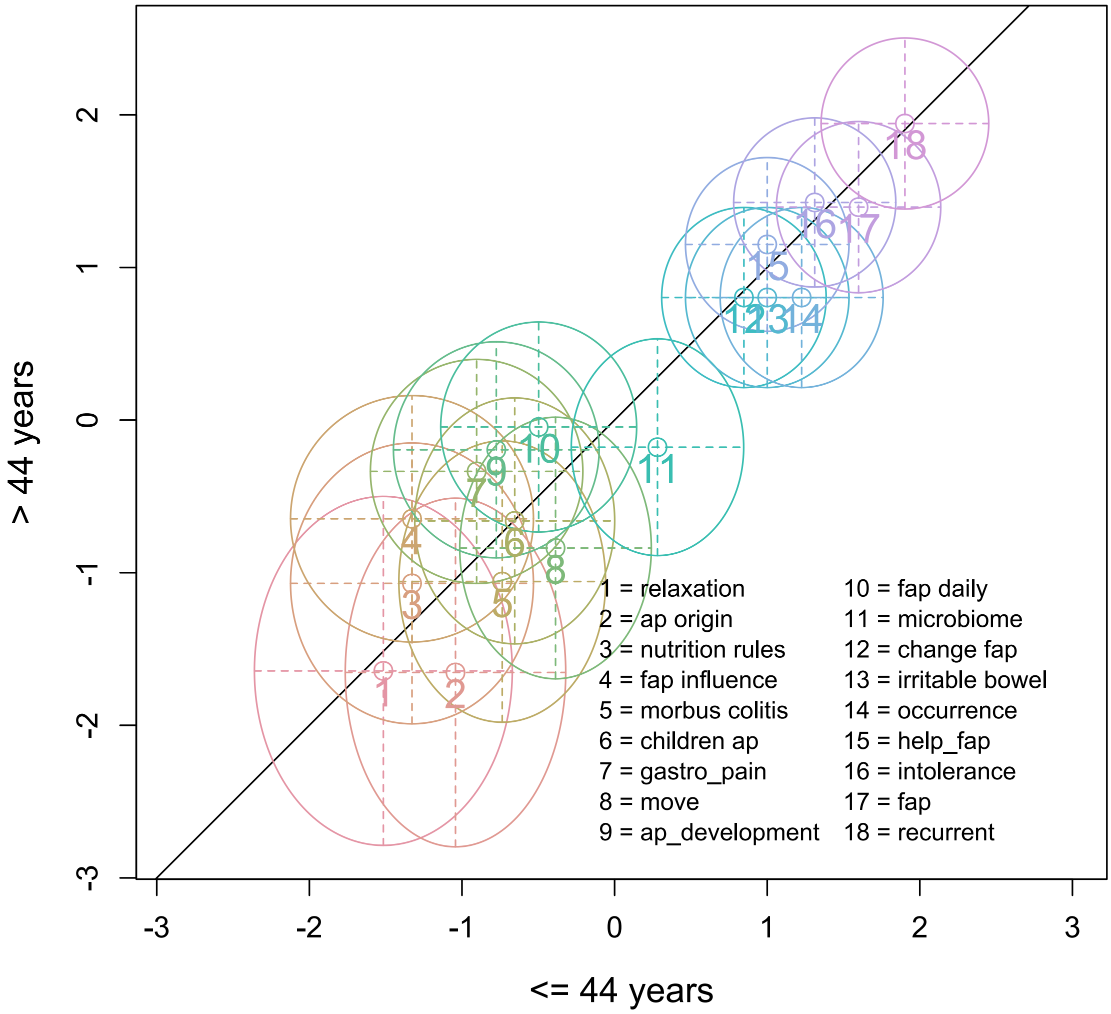

Figure S3. Visualization of Andersen's Likelihood-Ratio Test (LRT) with parent sample split by age group. The x-axis displays item parameters for parents younger or equal to 44 years of age; the y-axis displays item parameters for parents above 44 years. Zero indicates average difficulty, easier items below zero, and more difficult items above zero. The diagonal indicates parameter equality for both age groups. Circles around items show confidence intervals (CI). CI's overlapping with the diagonal indicate well-fitting items.

Figure S4

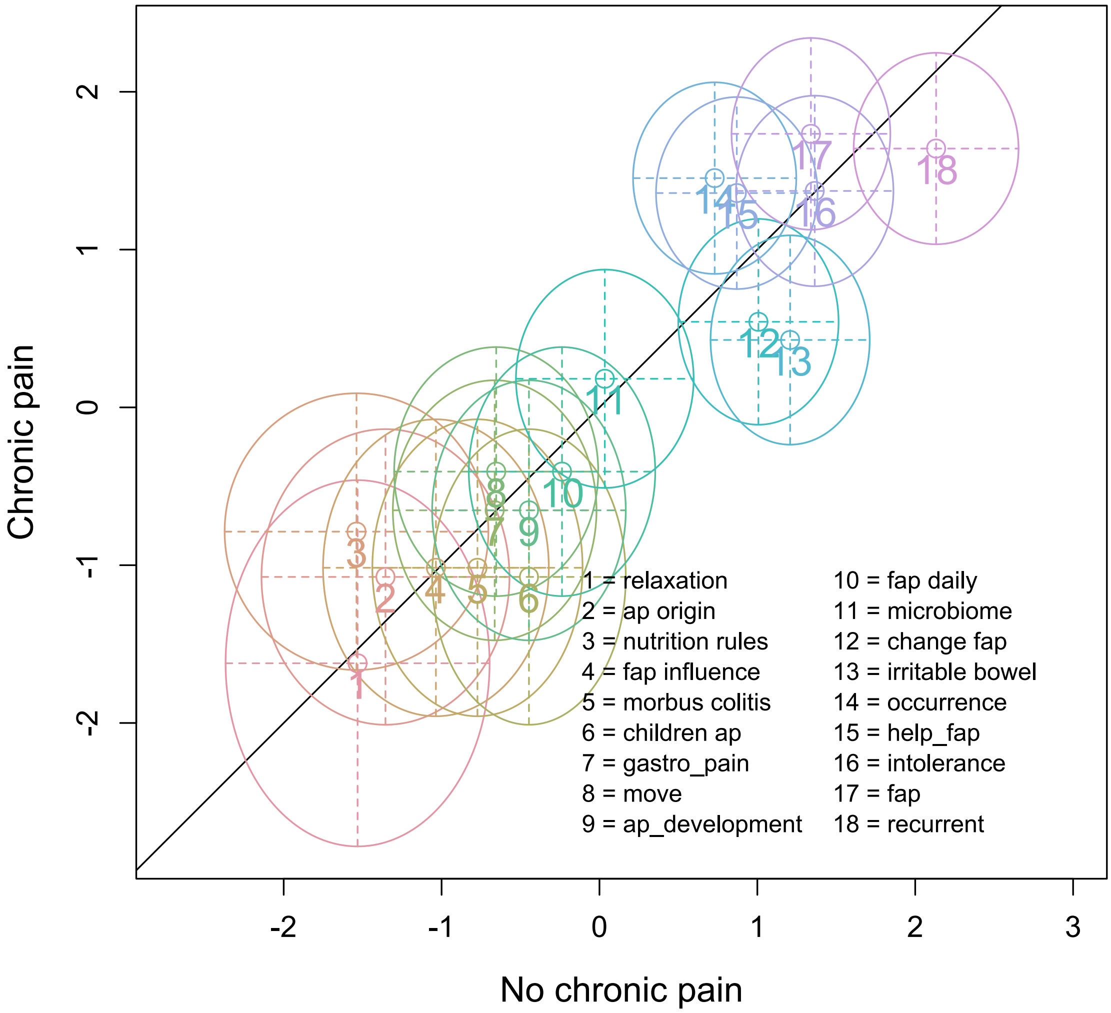

Figure S4. Visualization of Andersen's Likelihood-Ratio Test (LRT) with parent sample split by chronic pain group. The x-axis displays item parameters for parents without chronic pain; the y-axis displays item parameters for parents with chronic pain. Zero indicates average difficulty, easier items below zero, more difficult items above zero. The diagonal indicates good item fit. Circles around items show confidence intervals (CI). CI's overlapping with the diagonal indicate well-fitting items.

Figure S5

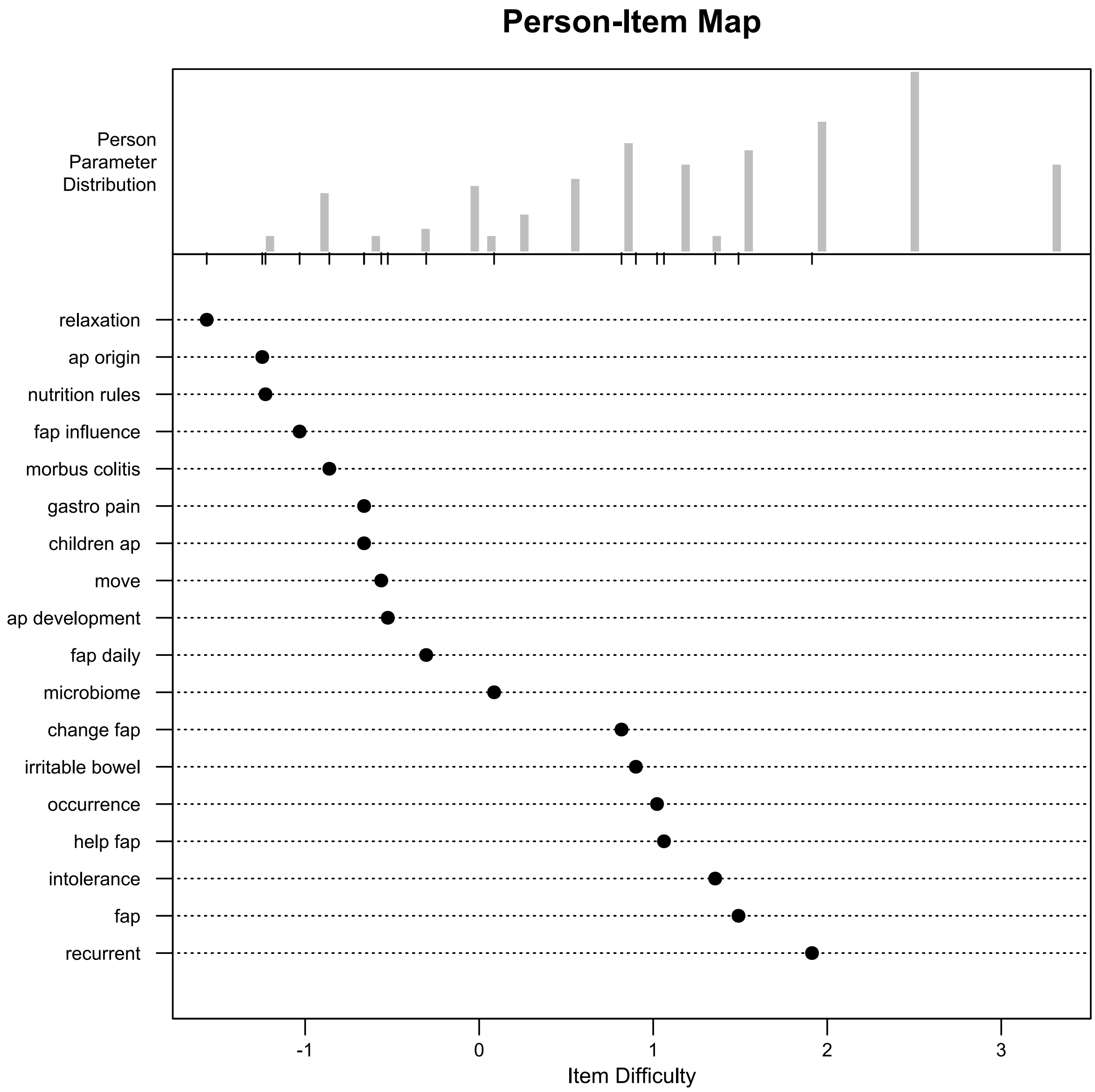

Figure S5. Person-Item map of the parent version of all A-PKQ items. The x-axis denotes item difficulty and is adjusted to the range of individual abilities (-1 to 3). Items are listed on the y-axis. The upper panel indicates the distribution of person parameters along the latent dimension. The lower panel depicts the item difficulties, where negative values in the x-axis represent easier items and positive values represent more difficult items.
